# Phylogenomic and Population Genomic Analyses of Ultraconserved Elements Reveal Deep Coalescence and Introgression Shaped Diversification Patterns in Lamprologine Cichlids of the Congo River

**DOI:** 10.1093/sysbio/syaf032

**Published:** 2025-05-13

**Authors:** Fernando Alda, S Elizabeth Alter, Naoko P Kurata, Prosanta Chakrabarty, Melanie L J Stiassny

**Affiliations:** Instituto de Investigación en Recursos Cinegéticos (IREC; CSIC-UCLM-JCCM), Ronda de Toledo 12, E-13071 Ciudad Real, Spain; Museum of Natural Science and Department of Biological Sciences, Louisiana State University, 119 Foster Hall, Baton Rouge, LA 70803, USA; Department of Ichthyology, American Museum of Natural History, 200 Central Park West, New York, NY 10024, USA; Department of Biology and Chemistry, California State University Monterey Bay, 100 Campus Center, Seaside, CA 93955, USA; Department of Ichthyology, American Museum of Natural History, 200 Central Park West, New York, NY 10024, USA; Department of Natural Resources and the Environment, Cornell University, 226 Mann Drive, 111 Fernow Hall, Ithaca, NY 14853, USA; Museum of Natural Science and Department of Biological Sciences, Louisiana State University, 119 Foster Hall, Baton Rouge, LA 70803, USA; Department of Ichthyology, American Museum of Natural History, 200 Central Park West, New York, NY 10024, USA; Department of Ichthyology, American Museum of Natural History, 200 Central Park West, New York, NY 10024, USA; The Sackler Institute for Comparative Genomics, American Museum of Natural History, 79th Street and Central park West, New York, NY 10024, USA

**Keywords:** African cichlids, Congo River, diversification, hybridization, Lamprologini, phylogenomics, UCEs, ultraconserved elements

## Abstract

Understanding the drivers of diversification is a central goal in evolutionary biology but can be challenging when lineages radiate quickly and/or hybridize frequently. Cichlids in the tribe Lamprologini, an exceptionally diverse clade found in the Congo basin, exemplify these issues: their evolutionary history has been difficult to untangle with previous data sets, particularly with regard to river-dwelling lineages in the genus *Lamprologus*. This clade notably includes the only known blind and depigmented cichlid, *Lamprologus lethops*. Here, we reconstructed the evolutionary, population, and biogeographic history of a *Lamprologus* clade from the Congo River by leveraging genomic data and sampling over 50 lamprologine species from the entire Lake Tanganyika radiation. This study provides the most comprehensive species-level coverage to date of the riverine taxa within this lacustrine-origin clade. We found that in the mid-late Pliocene, two lineages of Lake Tanganyika lamprologines independently colonized the Congo River, where they subsequently hybridized and diversified, forming the current monophyletic group of riverine *Lamprologus*. Our estimates for divergence time and introgression align with the region’s geological history and suggest rapid speciation in *Lamprologus* species from the Congo River marked by rapids-driven vicariance and water level fluctuations, and repeated episodes of secondary contact and reticulation. As a result of our analyses, we propose the taxonomic restriction of the genus *Lamprologus* to Congo River taxa only. The complex evolutionary history of this group—characterized by introgressive hybridization followed by a rapid series of isolation and reconnection—illustrates the multifaceted dynamics of speciation that have shaped the rich biodiversity of this region.

Accurately reconstructing phylogenies is crucial for understanding the patterns and processes underlying evolutionary diversification but can be challenging, especially when species share alleles due to incomplete lineage sorting (ILS) and/or introgressive hybridization (Koblmüller et al. 2010; Meyer et al. 2017). Both processes can produce patterns of DNA sequence similarity that may not accurately reflect population relationships; therefore, it is important to untangle their effects when phylogenetic discordance is observed (Kubatko and Degnan 2007; Edwards et al. 2016). This task, however, is not always straightforward and the footprints of ILS and hybridization on sequence data can vary across the genome and at different scales (e.g., temporal and taxonomic). Given these issues, exploring genome-wide data with both phylogenomic and population genomic approaches can be useful to interrogate discordant patterns within and between cellular genomes and in the reconstruction of progression of radiations across different timescales (Meyer et al. 2017; Ferrer Obiol et al. 2021; Svardal et al. 2021).

As a model group in speciation studies, African cichlids exemplify many of the evolutionary processes that can complicate phylogenetic reconstruction, including rapid speciation (e.g., Seehausen 2015; Irisarri et al. 2018; Astudillo-Clavijo et al. 2023), ILS (e.g., Koblmüller et al. 2010; Sturmbauer et al. 2010), and rampant hybridization (e.g., Keller et al. 2013; Meier et al. 2017), which has been suggested to play a key role in genotypic and phenotypic diversification during adaptive radiations (Meier et al. 2017, 2023; Malinsky et al. 2018a; Feller et al. 2020). The highly diverse tribe Lamprologini exemplifies both the phylogenetic challenges and the phenotypic and behavioral diversity of the family (Stiassny 1997). Although Lamprologini are endemic to Lake Tanganyika (LT), where they comprise about 40% of its 200+ described cichlid species (Ronco et al. 2020), at least 11 species are also found outside LT, most notably in the Congo River (CR; Stiassny and Alter 2021). Although CR *Lamprologus* comprise fewer described lineages compared with those in LT, these species are morphologically and ecologically diverse, with many exhibiting unique adaptations to rheophilic habitats and extreme hydrological conditions found in areas of the lower Congo River (LCR) such as *Lamprologus lethops*, a cryptophthalmic (i.e., “reduced eyed”) and depigmented cichlid; traits that are convergent with many cavefish species (Schobert et al. 2013; Aardema et al. 2020; Stiassny and Alter 2021).

The LCR comprises the last 350 km of the CR from Malebo Pool, a lake-like feature that separates the central Congo from the LCR, downstream to Boma (Democratic Republic of the Congo) (Fig. 1b). Along this short stretch, the river drops over 270 m, carrying the massive volume of the Congo Basin amassed in Malebo Pool, which then plunges into the Atlantic through narrow (<0.2 km) gorges and wide (>2 km) stretches, creating extremely turbulent flow, high-energy rapids, and deep underwater canyons (>220 m) (Robert 1946; Runge 2008; Jackson et al. 2009; Oberg et al. 2009; Stiassny and Alter 2021). Although it makes up only 2% of the area of the Congo Basin, the LCR harbors unparalleled richness (30% of all the CR cichlid species), endemism (25% of species), and morphological and ecological diversity of cichlids when compared with the rest of Africa’s rivers that are relatively depauperate in cichlid diversity (Roberts and Stewart 1976; Stiassny and Alter 2021). Thus, understanding the origin and diversification of the LCR freshwater fauna is key to answering questions about the evolutionary processes forming and maintaining riverine diversity hotspots. Although many studies have focused on the phylogenetic relationships and phylogeographic patterns of LT cichlids, including lamprologines (Sturmbauer et al. 1994, 2010; Day et al. 2007; Koblmüller et al. 2007, 2008; Nevado et al. 2009; Meyer et al. 2017; Irisarri et al. 2018; Schedel et al. 2019; Ronco et al. 2021; Astudillo-Clavijo et al. 2023), few analyses have included extensive sampling of CR species, and a comprehensive picture of the evolutionary history of lamprologines across the continent is lacking.

**Figure 1.**
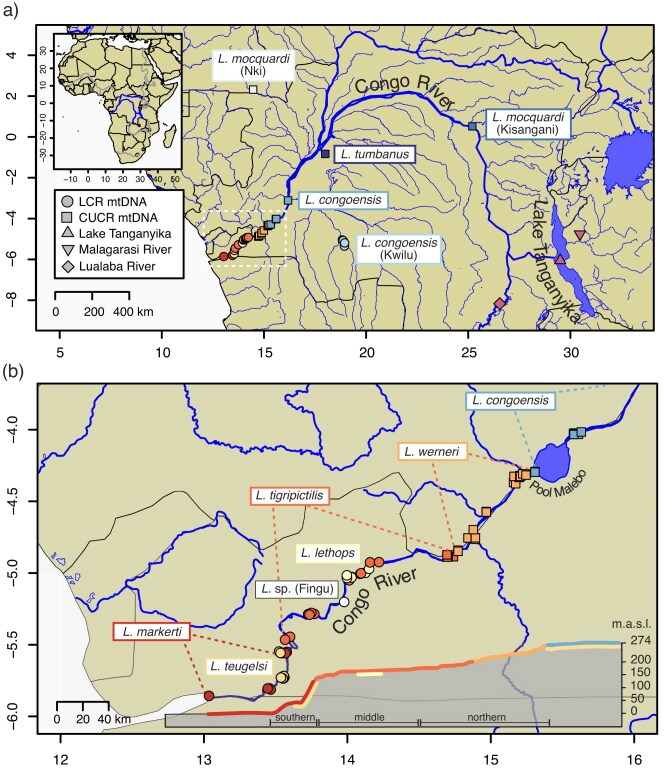
a) Map of sample collection sites in the CR basin. The inset map shows the location of the CR (in blue) and other major rivers (in grey) in Africa. The dashed rectangle indicates zoomed-in region below. b) Sampling locations in the LCR region. The colors correspond to species or clades as described in the text, and shapes (circles and squares) indicate whether that individual had an LCR or a CUCR mitochondrial haplotype. The cross-section diagram shows the elevational profile of the LCR (modified after Robert 1946) and the distributional ranges of *Lamprologus* species represented by colored lines.

Of the 11 described lamprologine species found outside of LT, 9 occur in the CR basin, all in the genus *Lamprologus* (Schelly and Stiassny 2004; Tougas and Stiassny 2014). The remaining two species belong to the genus *Telmatochromis* and are endemic to tributaries of LT, where *Telmatochromis devosi* inhabits the Malagarasi River, which flows into the lake’s eastern shore in Tanzania (Schelly et al. 2003), and *Telmatochromis salzburgeri* occurs in the Lufubu River, which drains into the southern shore of LT in Zambia (Indermaur et al. 2024). *Lamprologus congoensis* (the type species of the genus), and *L. mocquardi* are both widely distributed in the central and upper Congo River (CUCR). In contrast, *L. tumbanus* is endemic to Lake Tumba in the central Congo Basin, and *L. symoensi* is highly restricted to the Upemba Lakes region of the upper Lualaba (Schelly and Stiassny 2004). The remaining species are endemic to the LCR and adjacent habitats, most exhibiting narrow, parapatric distributions: *L. teugelsi* is known from Pool Malebo and from below the Inga rapids downstream in the LCR, *L. werneri* occurs in the upper section of the LCR, *L. tigripictilis* extends from the middle to the lower LCR including the Inga rapids, and *L. markerti* inhabits the lowest section of the LCR below the Inga rapids (Tougas and Stiassny 2014). The blind and depigmented *L. lethops* is only known from short stretches in the middle of the LCR, where it is believed to inhabit extremely deep and lightless canyons (Stiassny and Alter 2021) (Fig. 1). Kurata et al. (2022, 2024) used RADseq data to examine relationships and genetic structure within four LCR endemic species. They found support for the monophyly of each species and a deep phylogeographic split within the widely distributed LCR species *L. tigripictilis*, consistent with the “rapids-driven allopatry hypothesis.” They also found evidence of bidirectional gene flow, suggesting that individuals, under certain conditions, may be able to move both upstream and downstream. In addition to rapids acting as barriers, they proposed that recent Quaternary climatic changes impacted the diversification of these species: during glacial periods, reduced river discharge isolated populations and led to allopatric divergence, whereas wet interglacial periods facilitated migration of riverine fish. This finding challenges the long-held view of the CR as an ecosystem with relatively stable hydrological conditions (Roberts and Stewart 1976). In contrast, the severe water level fluctuations that East African lakes experienced in the last ∼1 myr have been linked to the dynamics of diversification of cichlid species flocks, and are considered one of the main drivers of isolation, secondary contact, and hybridization (Sturmbauer et al. 2001; Nevado et al. 2009; Koblmüller et al. 2010; Winkelmann et al. 2017). There remain questions about whether similar speciation mechanisms are acting in African riverine ecosystems.

Molecular studies of LCR fishes, although limited, agree on the vicariant role of high-energy hydraulics and complex bathymetry, promoting the isolation of populations and processes of microallopatric speciation (Markert et al. 2010; Alter et al. 2017; Kurata et al. 2022). Geomorphological features, such as Malebo Pool, or the massive downstream Inga Rapids divide the LCR into three biogeographical regions (Fig. 1b; Robert 1946) that represent areas of endemism and shared population genetic breaks (Markert et al. 2010; Schwarzer et al. 2011). These extreme conditions have also resulted in convergent adaptations such as cryptophthalmic phenotypes in at least 9 LCR endemic species across lineages of spiny eels (Mastacembelidae), cichlids (Cichlidae), elephant-nose fishes (Mormyridae), and catfishes (Claroteidae, Clariidae) (Alter et al. 2015). Consistent with geological evidence suggesting a young age for the contemporary hydrological conditions of the LCR, all cichlid lineages, and other fish taxa (e.g., killifishes, Collier et al. 2009; catfishes, Day et al. 2013; spiny eels, Alter et al. 2015), have arrived recently at ≤3–5 Ma to the CR; the earliest being the chromidotilapiines (Schwarzer et al. 2015; Alter et al. 2017), followed by haplochromines originating in the east African lakes (Schwarzer et al. 2012b), and most recently the lamprologines dispersing from LT to the proto-CR (Day et al. 2007; Sturmbauer et al. 2010; Schedel et al. 2019). Interestingly, many lineages show multiple independent colonizations (Schwarzer et al. 2011; Alter et al. 2015, 2017). Additionally, iterative periods of connectivity among basins may have shaped species distributions and provided opportunities for hybridization following secondary contact (Schwarzer et al. 2011, 2012a, 2012b).

A variety of molecular markers have been used to analyze lamprologine relationships, and the monophyly of the tribe is firmly established by both molecular (Takahashi et al. 1998; Meyer et al. 2015; Ronco et al. 2021; Astudillo-Clavijo et al. 2023) and morphological (Stiassny 1997) data, but the composition and relationships among genera are far less clear and many currently recognized genera appear to be polyphyletic. Although recent studies consistently find that sampled CR species nest within the LT radiation, there is considerably less agreement about their placement in subclades. Sturmbauer et al. (2010) (using mtDNA and AFLP) and Day et al. (2007) (mtDNA) placed *L. congoensis* and *L. teugelsi* within a subclade of *Telmatochromis* as the sister group to *T. vittatus*, although they did not include any other riverine species. In contrast, the taxonomically complete mitogenomic study of Jimenez et al. (2024) and that of Schedel et al. (2019) found that CR species were non-monophyletic, grouping in two lineages each, respectively, more closely related to other LT lamprologines. Ronco et al. (2021), using whole-genome data from nearly all LT endemic cichlids, consistently recovered *L. tigripictilis* (the only CR *Lamprologus* included in that study) within a clade containing *Neolamprologus christyi, N. cunningtoni, N. modestus, N. mondabu, N. tetracanthus*, and others. More recently, Astudillo-Clavijo et al. (2023) reconstructed relationships of African cichlids (Pseudocrenilabrinae) using hundreds of single-copy exons and proposed monophyly of CR *Lamprologus*, monophyly of the LCR species within that clade, and a sister relationship of these clades with *N. mondabu*. However, their analysis recovered relatively weak bootstrap and posterior probability values for a monophyletic LCR clade. Indeed, in all previous studies, the position of CR *Lamprologus* and their relationships have been recovered with low support.

Several challenges remain in resolving the evolutionary history of CR *Lamprologus*. Many areas of the basin have limited access, so current sampling of species may not fully represent their geographic ranges, and there are likely as yet unidentified lineages (Stiassny and Alter 2021). Additionally, given previous challenges in resolving deeper nodes, even using hundreds of exons (Astudillo-Clavijo et al. 2023), it seems likely that CR *Lamprologus* have radiated rapidly and/or experienced periods of hybridization, as has been demonstrated in LT Lamprologini (Irisarri et al. 2018; Astudillo-Clavijo et al. 2023); which may explain the extensive mitonuclear discordance that has been documented (Schedel et al. 2019; Jimenez et al. 2024).

In this study, we aim to reconstruct the evolutionary history of CR *Lamprologus* using genomic data (ultraconserved elements [UCEs]) and geographical sampling focused on the riverine taxa. Specific goals of the study are 1) to test the monophyly of the CR species and two recovered subgroupings: central and upper Congo River basin species (CUCR, including Lake Tumba) and species endemic to the LCR (defined as downstream from Pool Malebo); 2) to reconstruct phylogenetic relationships and population structure within CR *Lamprologus* in the context of biogeographic distributions, including the identification of the sister clade of the enigmatic *L. lethops*; 3) estimate divergence times between the major clades; and 4) examine the mechanisms driving the CR radiation including allopatric divergence and hybridization.

## Materials and Methods

### Taxon Sampling and UCE Data Acquisition

Taxon sampling comprises 167 samples of 57 described cichlids representing 13 genera of the East African Radiation (EAR). The ingroup (Lamprologini) includes 162 specimens spanning 52 described species including representation of all currently recognized genera. Of these samples, 99 belonged to 8 of the 9 described species of CR *Lamprologus* (exclusive of *L. symoensi* for which no tissues were available, but see Taxonomy and Nomenclature section). The outgroup is composed of representatives of four genera of haplochromine cichlids (H-lineage, sensu Meyer et al. 2015) and one sample of *Boulengerochromis microlepis* (tribe Boulengerochromini), representing the crown of the EAR clade used to root the tree. Samples were collected by the authors, loaned from natural history collections, or data mined from online repositories (Fig. 1 and Supplementary Table S1a).

We constructed genomic libraries from 159 samples and enriched them using a set of 2001 probes (Actinopts-UCE-0.5Kv1), targeting 500 UCE loci across Actinopterygii (Faircloth et al. 2013), and following methods in http://ultraconserved.org with slight modifications (Burress et al. 2018; full details of laboratory methods for library preparation, hybrid-capture reactions, and bioinformatic pipeline are provided in Appendix A of the Supplementary Materials). Additionally, we extracted the same UCEs *in silico* for three genome-enabled species *Neolamprologus brichardi* (RefSeq: GCF_000239395.1) (Brawand et al. 2014), *L. lethops* (*n* = 5), and *L. tigripictilis* (*n* = 1), and one undescribed cryptophthalmic and depigmented LCR species, informally designated *L*. sp. “Fingu” (*n* = 1) (see Taxonomy and Nomenclature section, Supplementary Table S1a). Full details describing the bioinformatic pipeline are provided in Supplementary Material Appendix A. Sequence reads are deposited in the NCBI sequence read archive (SRA) under BioProject accession PRJNA1097814.

### Phylogenomic Analysis

We filtered the entire set of aligned UCEs and created an incomplete matrix containing data for at least 75% of the samples and removed those loci with over 70% of missing data. We computed alignment statistics and the number of informative sites across alignments and concatenated them into a PHYLIP supermatrix.

We used the concatenated alignment to estimate a maximum likelihood phylogenetic hypothesis in IQ-TREE 2 (Minh et al. 2020), selecting the best substitution model for each UCE and partition scheme using ModelFinder (Kalyaanamoorthy et al. 2017) implemented in IQ-TREE 2, followed by tree inference in the same run using the option -m MFP. We evaluated nodal support using Ultrafast bootstrap (UFBoot2) (Kalyaanamoorthy et al. 2017; Hoang et al. 2018).

To account for heterogeneity across gene trees, we inferred a multispecies coalescent (MSC) species tree using the summary coalescent-based method of ASTRAL-III (Zhang et al. 2018). ASTRAL-III uses a quartet-based approach to find the species tree that shares the maximum number of quartets within a set of gene trees. For this purpose, we estimated gene trees for each UCE locus in IQ-TREE 2 and used them as input for ASTRAL-III. Branch support of the species tree was assessed using local posterior probabilities (LPPs; Sayyari and Mirarab 2016). Like all summary methods, ASTRAL-III relies on accurately estimated gene trees, and it is therefore sensitive to poorly supported branches (Zhang et al. 2018). Therefore, we repeated the MSC species tree analysis using the same set of input gene trees after removing branches with nodal support of BS = 0 using Newick Utilities 1.6 (Junier et al. 2010).

Disagreement between individual gene trees and the MSC species tree was explored using normalized quartet scores (NQS), representing the proportion of reconstructed gene trees that agree with the reference species tree divided by the total number of quartets. Scores range between 0 and 1, where 0 indicates that none of the quartets agree between the species and the gene tree, suggesting poor accuracy, and 1 indicates that all quartets agree, suggesting a higher level of accuracy in the reconstruction. We annotated the species tree in ASTRAL-III using the option -t 16 to calculate the NQS across the branches of the preferred species tree topology and the two alternative topologies. For this analysis, we used the MSC species tree with all the species included but focused on the relationships among the CR *Lamprologus*. Finally, we performed a polytomy test (option -t 10 in ASTRAL-III) to evaluate whether certain branches in the species tree, under MSC assumptions, should be replaced by (hard) polytomies (Sayyari and Mirarab 2018).

Given the low support and uncertainty of some relationships among major clades in the ML and MSC species trees, we carried out tests for significant topological differences between the inferred ML tree and alternative phylogenetic hypotheses. As we were mostly interested in the relationships among CR species, we used IQ-TREE 2 to infer alternative topologies by constraining the relationships of *L. lethops* and *L. teugelsi* relative to the CR *Lamprologus* clade, the LCR clade, and by constraining the monophyly of *L. mocquardi* and *L. congoensis* while not making assumptions about other relationships (Supplementary Table S3). We then performed approximate unbiased (AU; Shimodaira 2002) topology tests between the constrained and the unconstrained ML topologies using options -zb 10,000 -au in IQ-TREE 2.

### Divergence Time Estimation

We constructed a timetree in BEAST2 v.2.7.6 (Bouckaert et al. 2019) to estimate divergence times for Lamprologini based on one fossil-based prior and one secondary calibration. The fossil calibration is *Tugenchromis pickfordi* (Altner et al. 2017) from the Upper Miocene (9–10 Ma) and placed at the node of the most recent common ancestor (MRCA) of the H-lineage and the Lamprologini, given its affinity to these tribes (Lognormal distribution with mean = 0.00 Ma, offset = 9.00, SD = 1.8, 95% quantiles: 9.3–43.2 Ma; Genner et al. 2007; Irisarri et al. 2018). We constrained the maximum age with a secondary calibration representing the MRCA of the EAR (Normal distribution with mean = 13.7 Ma, sigma = 3.4, 95% quantiles: 8.11–19.3 Ma; Irisarri et al. 2018).

To reduce computational demands, we pruned our UCE data set to 61 tips representing one specimen per taxon (for taxa with more than one sample sequenced, we selected the most data-complete sample; Supplementary Table S1a) and subdivided the data set into four alignments: three with 100 random loci and one containing 109 random loci. We concatenated each subset (mean subset length = 44,624.5 bp, SD = 2972.94) and partitioned them following the best scheme estimated by ModelFinder in IQ-TREE 2. We used an HKY substitution model with 4 gamma rate categories and set the base frequencies to “empirical” with unlinked site model parameters. We used a relaxed lognormal clock model, a Yule tree prior, and fixed the tree topology to the ML hypothesis by switching off the subtreeSlide, narrowExchange, wideExchange, and wilsonBalding operators, setting them to zero. Clock model parameters were linked across partitions.

We ran each subset of ∼100 loci twice with a Markov chain Monte Carlo (MCMC) chain length of 1 × 10^8^ generations, a log sampling frequency of 1 × 10^5^ generations, and a tree sampling frequency of 1 × 10^4^ generations. To evaluate sampling independence and autocorrelation within MCMC chains, we used Tracer v.1.7.1 (Rambaut et al. 2018), ensuring effective sampling sizes (ESS) exceeded 200. Additionally, we assessed convergence across runs using TreeStat v.1.2 http://tree.bio.ed.ac.uk/software/treestat/ by confirming that estimated node ages were consistent across all replicates and alignment subsets. We then combined the posterior distribution of trees for all 8 runs following a 10% burn-in in LogCombiner v.2.7.6, and summarized the resulting file using TreeAnnotator v.2.7.6 (Bouckaert et al. 2019).

### Species Networks and Hybridization Analysis

In addition to investigating bifurcating evolutionary models, we employed a phylogenetic network method to visualize reticulate relationships among taxa and to identify incongruence among gene trees on the species tree. We constructed a neighbor-net analysis using ML distances inferred from concatenated UCE sequence data and performed 1000 bootstrap replicates to assess support using SplitsTree v.4.12.3 (Huson and Bryant 2006). We also measured whether the data fit well into a bifurcating tree model using the delta score (Holland et al. 2002) calculated for the whole data set and for each group of taxa separately. This measure scores taxa from 0 to 1, where a perfectly bifurcating tree will have a score of zero and a perfect network will have a score of 1.

We tested the hypothesis that phylogenetic discordance is due to introgressive hybridization between species. We used the Patterson’s *D*-statistic (Patterson et al. 2012) to identify an excess of shared derived polymorphisms between non-sister species, indicated by the relative abundance of ABBA and BABA SNP patterns across all possible 4-taxon trees with three populations or species and an outgroup with the relationship (((P1, P2), P3) O). Alternatively, under the null hypothesis of ILS without gene flow, ABBA and BABA patterns should be equally frequent (Green et al. 2010; Durand et al. 2011). For each species trio, we also calculated the admixture fraction or *f_4_*-ratio (Patterson et al. 2012), which estimates the proportion of introgressed material in an admixed population. For easier interpretation, *f*_4_-ratios were summarized using the *f*-branch statistic method that provides for each branch a summary of the excess allele sharing with its non-sister species or population (Malinsky et al. 2018a). We used the Dsuite software package (Malinsky et al. 2021) to calculate *D*- and *f*-statistics for all the possible trios of CR *Lamprologus* (outgroup fixed to *T. devosi* and *N. christyi*; Supplementary Table S1a) using the functions Dtrios and Fbranch. Because these estimates rely on the phylogenies used being correct, we carried out the analysis independently for ML and MSC topologies. We determined statistical significance using *Z*-scores associated with each *D*-statistic (|Z| > 3) and their respective *P* values (*P* < 0.01).

### Phylogenetic Analysis of Mitochondrial DNA

We obtained sequences of the mitochondrial gene NADH dehydrogenase subunit 2 (ND2) by mapping cleaned Illumina sequencing reads of all our samples to the ND2 gene of *L. tigripictilis* (GenBank number OQ862832; Jimenez et al. 2024) using the BBMap aligner (https://sourceforge.net/projects/bbmap/) as implemented in Geneious Prime 2023.0.4. Only contigs with more than 5× mean coverage were retained and we aligned the consensus sequences (accession numbers: PQ307172–PQ307257) with ND2 sequences of Pseudocrenilabrinae available in GenBank (Supplementary Table S1b).

We reconstructed ND2 phylogenetic trees using ML and Bayesian inference (BI) methods and estimated the evolutionary model that best fit the mitochondrial data using ModelFinder under the Bayesian Information Criterion (TIM2 + F + I + R4), inferred the best ML tree, and evaluated nodal support with 1000 replicates of Ultrafast bootstrap in IQ-TREE 2 (option -m MFP). We performed Bayesian analysis in MrBayes v.3.2.7 (Ronquist et al. 2012) using the GTR evolutionary model, running 2 simultaneous and independent sets of 4 MCMC of 1 × 10^6^ generations each, sampling trees every 5000 generations. We assessed convergence of the MCMC chains by checking the average standard deviation of split frequencies among MCMC runs (ASDSF < 0.01) and the ESS across all parameters (ESS > 200) in Tracer v.1.7.1 (Rambaut et al. 2018). We discarded the first 25% of samples as burn-in and calculated posterior probabilities of nodes to evaluate support for the phylogenetic hypothesis.

We also constructed a calibrated time tree for the mitochondrial ND2 gene data using BEAST2 v.2.7.6. We used the GTR model and estimated substitution rates to infer the phylogenetic tree, to which we applied a lognormal relaxed molecular clock, assuming a Yule speciation model and the same calibration scheme as in the UCE tree. We ran the MCMC for 1 × 10^8^ generations, sampling every 1 × 10^4^ generations, and confirmed convergence in Tracer v.1.7.1. Finally, we discarded 10% of the sampled trees as burn-in and summarized the remnant posterior distribution of trees into a maximum clade credibility tree using TreeAnnotator v.2.7.6.

### Population Genomic Analysis

We extracted SNPs from the UCE data of 93 samples of CR *Lamprologus* (Supplementary Table S1a) to analyze diversity and structure from a population genomic perspective. Following indexing, alignment, and quality control of raw sequence data, we exported the passing SNPs into a VCF file and removed individuals missing more than 75% of SNPs and all loci missing more than 50% of individuals, resulting in a data set of 11,189 SNPs. We also created a second VCF file by selecting one random SNP per locus, resulting in 427 SNPs (full details of the bioinformatic pipeline are provided in Appendix A of Supplementary Materials).

To describe genetic variation among CR *Lamprologus* without making a priori assumptions about existing groups, we first conducted a principal component analysis (PCA) using the data set with one random SNP per locus. We converted the VCF file into a genind object using the function “vcfR2genind” in R package vcfR (Knaus and Grünwald 2017) and conducted a PCA on these genotypes using “dudi.pca” in ade4 (Dray and Dufour 2007), keeping 20 axes centered by the mean. Secondly, we estimated individual admixture coefficients and inferred the number of ancestral populations (*K*) from which they may be derived, using sNMF, a method based on sparse non-negative matrix factorization (NMF) and least-squares optimization (Frichot et al. 2014), implemented in the R package LEA (Frichot and François 2015). Unlike traditional likelihood-based approaches like STRUCTURE or ADMIXTURE, sNMF (along with PCA) relaxes common population genetic assumptions, such as Hardy–Weinberg equilibrium, making it suitable for analyzing both inbred and outbred ancestral populations. Additionally, sNMF performs more efficiently, with significantly faster runtimes compared with likelihood-based methods (Frichot et al. 2014).

We converted the VCF file of the genotypic matrix with one random SNP into a geno object using “vcf2geno” in vcfR and ran the function “snmf,” assuming *K* ancestral populations from *K* = 1–14, with 100 repetitions per *K*, and regularization parameter of α = 10. We then used the entropy criterion (i.e., the smallest value of cross-entropy) to choose the number of ancestral populations that best explain the genotypic data and calculated individual ancestry proportions.

Finally, we inferred recent shared ancestry among species using fineRADstructure (Malinsky et al. 2018b), which uses haplotype linkage information to calculate a co-ancestry matrix based on the most recent coalescence among sampled individuals. We used the genotype matrix with all extracted SNPs transformed into a Tag Haplotype Matrix using the function ‘hapsFromVCF’ in RADpainter, included in fineRADstructure. We assumed perfect linkage among SNPs within each UCE locus and frequent recombination between loci. We calculated the co-ancestry matrix using the function “paint” in RADpainter and assigned individuals to populations using fineSTRUCTURE by running the MCMC for 1 × 10^5^ generations following an initial burn-in of 1 × 10^5^ generations. We visualized and plotted the results using R scripts in fineRADstructurePlot.R (available at https://github.com/millanek/fineRADstructure).

## Results

We collected 499 UCE loci from 159 samples using a hybrid capture and enrichment protocol, and from 8 complete genomes *in silico*. We sequenced 241.84 million reads with an average of 1,550,283.03 (±753,695.38 SD) reads per sample (min–max = 411,292–4,585,437), which were assembled into 44.1 million contigs (mean = 265,712.35 contigs per sample; min–max = 4781–886,880). On average we recovered 397.92 (±90.15 SD) UCE loci per sample (min–max = 93–492) that averaged 847.8 bp long after alignment (min–max = 101–2293) (Supplementary Table S2). Each locus was sequenced on average for 128.75 individuals (82.53%), but some loci were as low as 3 individuals. Therefore, our alignments only used loci that included at least 75% of the individuals (i.e., a minimum of 125 individuals per locus). For the phylogenomic analysis, we also removed specimens missing more than 70% of loci (minimum of 349 UCEs). The concatenated alignment included 164 individuals at 409 UCE loci for a total of 181,498 bp. The full SNP data set included 93 samples of CR *Lamprologus* and 11,189 SNPs in 427 loci (Supplementary Table S1a).

### Phylogenomic Analysis

**Figure 2.**
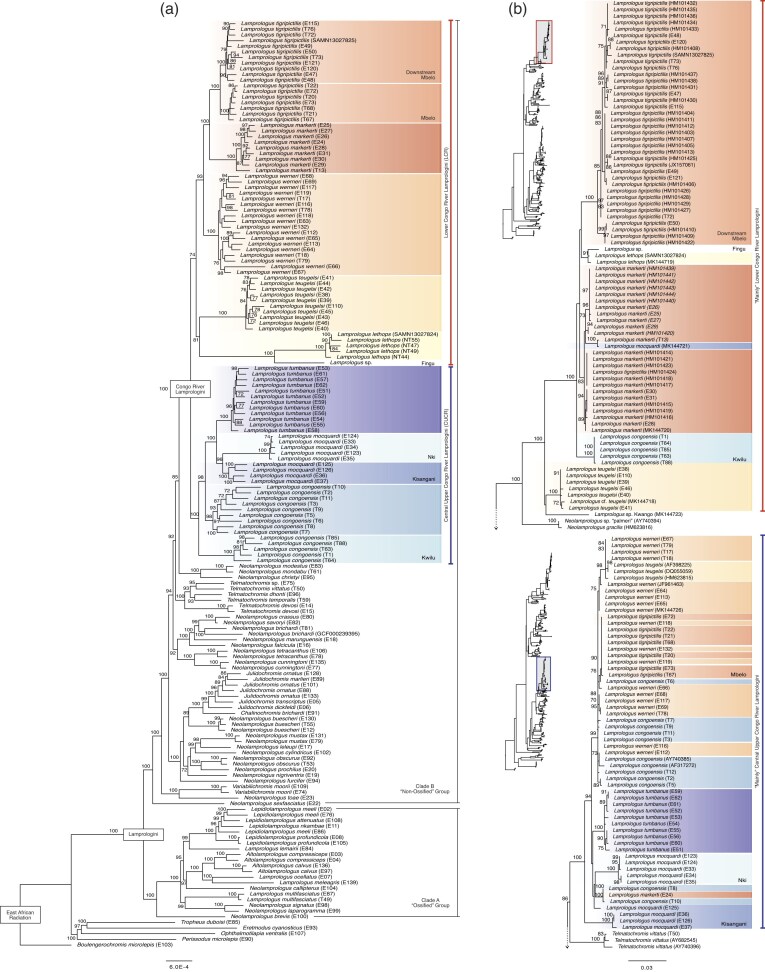
Maximum likelihood phylogenetic hypotheses of Lamprologini based on a) the concatenated analysis of UCEs and b) mitochondrial gene ND2 sequences using IQ-TREE2. Species and major clades of CR *Lamprologus* are highlighted with colors. For clarity, only bootstrap values ≥70 are shown, and in the mitochondrial tree we only show the CR *Lamprologus* clades. The grey boxes in the inset trees show the positions of these clades in the full tree (complete mitochondrial ND2 gene trees in Supplementary Figs. S1 and S2).

**Figure 3.**
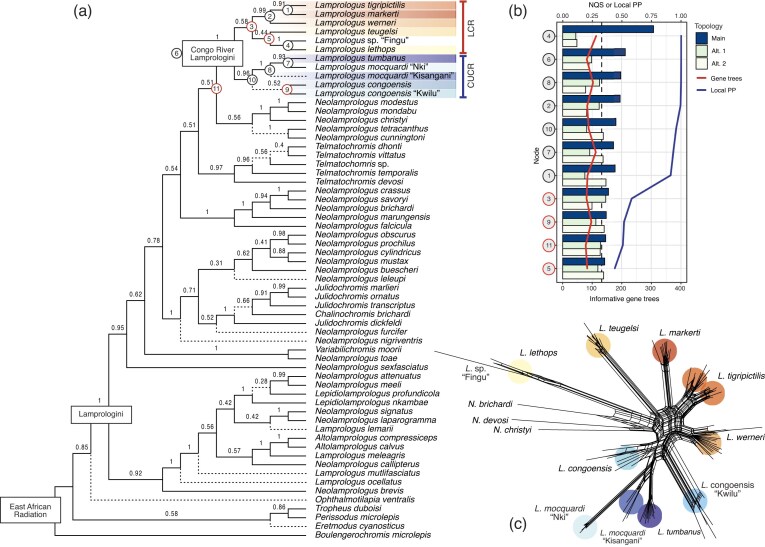
a) Species tree of Lamprologini inferred in ASTRAL-III based on 409 UCE gene trees. The numbers above branches indicate support (LPP) for the adjacent node. The dashed branches represent disagreement between the species tree and the concatenated ML tree. Species of CR *Lamprologus* species are highlighted with colors (LCR: lower Congo River; CUCR: central and upper Congo River species) and their nodes are numbered (in circles). The red circles indicate that for these nodes we failed to reject the null hypothesis of the polytomy test of nonzero length branches. b) Graphical representation of the NQS recovered for the three possible alternative topologies around each branch of the riverine *Lamprologus* clade. The dashed, black line indicates the polytomy limit of the quartet scores (∼0.333). The blue and red lines indicate the LPP and the number of informative gene trees for each node. c) Phylogenetic network of the riverine *Lamprologus* species constructed in SplitsTree.

Concatenated ML and MSC phylogenomic trees were largely congruent and recovered the same major relationships (Figs. 2 and 3a). In common with previous studies with over 50% sampling of LT Lamprologini (Day et al. 2007; Sturmbauer et al. 2010; Ronco et al. 2021), we inferred two major lineages. One (Clade A, Fig. 2a, BS = 100, LPP = 0.92) is composed exclusively of LT endemics, including all sampled species of *Lepidiolamprologus* and *Altolamprologus*, some species of *Lamprologus* (*L. callipterus, L. lemairii, L. meleagris, L. multifasciatus, L. ocellatus*) and of *Neolamprologus* (*N. brevis, N. laparogramma, N. signatus*). This clade, informally named the “ossified group” or Stiassnia by Day et al. (2007), is characterized morphologically by the presence of a sesamoid ossification (labial bone) suspended in the labial ligament of the lower jaw (Stiassny 1997, figure 13). Although internal relationships within the clade vary markedly between studies, they are consistent with the present study in recovering both *Lepidiolamprologus* and *Altolamprologus* as monophyletic.

The second major lineage (Clade B, Fig. 2a, BS = 100, LPP = 0.95) includes our sampling of the LT genera *Chalinochromis, Julidochromis, Telmatochromis, Variabilichromis*, numerous *Neolamprologus*, and all sampled members of CR *Lamprologus* (Figs. 2a and 3a). This assemblage has informally been named the “non-ossified group (Day et al. 2007),” and although recognized in previous molecular studies is currently lacking morphological character support. Again, resolution of generic limits and species relationships among the LT species included in this clade varies greatly between studies. However, noteworthy is our resolution of the morphologically derived genera *Julidochromis* as monophyletic and as the sister group to *Chalinochromis*. Additionally, *Telmatochromis* is recovered as monophyletic, with the riverine *T. devosi* (Malagarasi basin) as the sister lineage to all other sampled members of the genus. Both *Julidochromis* and *Chalinochromis* were found to be non-monophyletic by Day et al. (2007), Sturmbauer et al. (2010), and Ronco et al. (2021); albeit with differing topologies. Similarly, our placement of a small group of LT *Neolamprologus* (*N. christyi, N. cunningtoni, N. modestus, N. mondabu*, and *N. tetracanthus*) as the sister group to the CR *Lamprologus* radiation differs markedly from the aforementioned multilocus studies. However, it aligns with the phylogenomic analyses of Ronco et al. (2021) and Astudillo-Clavijo et al. (2023). Although the latter study had limited taxon coverage, it also recovered *N. mondabu* as the sister lineage to the CR *Lamprologus*.

The central focus of the present study is the clade comprised of all sampled CR *Lamprologus* (BS = 100, LPP = 1). In the ML tree, this clade is divided into two subclades with moderate support (Fig. 2a). One includes all CUCR species (BS = 100), with *L. tumbanus* resolved as the sister species to *L. mocquardi*, which showed strong geographical structure between individuals from Nki (Dja River, Cameroon) and from Kisangani (mainstem middle Congo River, Democratic Republic of the Congo) forming two reciprocally monophyletic groups (BS = 96); this group is sister to *L. congoensis* from the Congo main channel sampled from Pool Malebo to Bolobo (Democratic Republic of the Congo) (BS = 100). Individuals identified as *L. congoensis* (*L. congoensis* “Kwilu”) from the Kwilu River, a Kasai tributary draining into the Kwa upstream of Pool Malebo (Fig. 1a), were resolved as the sister group to all other CUCR species (BS = 100). The second subclade includes all LCR species (BS = 74). *Lamprologus tigripictilis* displayed geographical structuring with individuals from Mbelo (Republic of the Congo) differentiated from individuals sampled from downstream of Mbelo to the Inga rapids. *Lamprologus markerti* and *L. tigripictilis* were recovered as sister groups (BS = 100), and *L. werneri* was resolved as the sister group to that lineage (BS = 93). The closest relative of the blind cichlid *L. lethops* was found to be an undescribed *Lamprologus* sp. collected at Fingu, a locality near Mampambala Village (Republic of the Congo) on the right bank of the Congo River (Fig. 1b) (BS = 100). Individuals of *L. teugelsi* (from Nziya and Yalala, Democratic Republic of the Congo) were recovered as the sister group to the *L. lethops *+ *L*. sp. “Fingu” pair, although these were joined by long branches and a short internode without strong support (BS = 81).

The MSC species tree recovers the same two subclades but with lower support: the CUCR group with LPP = 0.96, and the LCR group with LPP = 0.58 (Fig. 3a). Within subclades, relationships among species were mostly recovered as they were in the ML tree, but also with lower support. A few exceptions were seen in the CUCR group, where *L. mocquardi* from Kisangani was found as the sister species to *L. tumbanus* (LPP = 0.93), and that pair was recovered as sister to *L. mocquardi* from Nki (LPP = 1). In the LCR group, *L. lethops* was resolved as sister to *L*. sp. “Fingu” with high support (LPP = 1) but with a poorly supported sister relationship to *L. teugelsi* (LPP = 0.44). As in the ML tree, *L. tigripictilis* was sister to *L. markerti* (LPP = 0.91) and together they formed the sister lineage to *L. werneri* (LPP = 0.99). However, polytomy tests failed to reject the null hypothesis of zero-branch length (i.e., a polytomy) for relationships within the (*L. teugelsi*, [*L. lethops, L*. sp. “Fingu”]) clade (*P *= 0.782), between them and the (*L. werneri*, [*L. tigripictilis, L. markerti*]) clade (*P *= 0.246), and between *L. congoensis* and *L. congoensis* “Kwilu” (*P *= 0.524) (Fig. 3a,b).

Timetree analysis estimated a mean age for the EAR radiation at 10.62 Ma (95% HPD 9.03–14.68 Ma). The MRCA of Lamprologini began diversification in the late Miocene (∼7.12 Ma; 95% HPD 5.39–10.12 Ma), with the two major clades diversifying around the Miocene–Pliocene transition (∼6.07 Ma; 95% HPD 4.42–8.74 Ma and ∼6.39 Ma; 95% HPD 4.70–9.11 Ma). The split between the LT and CR *Lamprologus* appears to have occurred in the mid-Pliocene, around 3.7 Ma (95% HPD 2.5–5.55 Ma), with the diversification of the LCR and CUCR clades occurring shortly thereafter (∼3.36 Ma; 95% HPD 2.25–5.09 Ma and ∼3.17 Ma; 95% HPD 2.1–4.84 Ma, respectively). Similarly, the mean ages of the MRCA of the CR *Lamprologus* indicate that all the species diverged rapidly during the first half of the Pleistocene (1.54–2.95 Ma).

The exploration of NQS across the species tree suggests moderate to high levels of gene tree discordance (Fig. 3b and Supplementary Fig. S3). Overall NQS was 0.659, indicating that 65.9% of gene tree quartets (1.267 × 10^9^ quartets) are found in the species tree. As has been evidenced before, high nodal support is not necessarily correlated with high congruity. In our species tree, well-supported nodes (i.e., LPP > 0.9) had an average NQS of 0.522 (SD = 0.104, *n* = 33). Although some nodes showed high congruity, such as the divergence between clades A and B of Lamprologini (NQS t1 = 0.74, t2 = 0.14, t3 = 0.11), other nodes showed only moderate congruity, such as for the monophyly of CR *Lamprologus* (NQS t1 = 0.53, t2 = 0.24, t3 = 0.22). Notably, the second highest NQS in the species tree corresponds to the sister relationship between *L. lethops* and *L*. sp. “Fingu” (NQS t1 = 0.77, t2 = 0.11, t3 = 0.12) (Fig. 3b).

Some nodes with low support showed alternative topologies with similar NQS, suggesting that ILS may be causing gene tree disagreement. For example, the split between the CR *Lamprologus* and the (*N. christyi*, (*N. modestus, N. mondabu*)) clade (NQS t1 = 0.36, t2 = 0.32, t3 = 0.31). Furthermore, the polytomy test for that internode was non-significant (*P* ≥ 0.294), thus failing to reject the null hypothesis of a zero-branch length. Notably, these relationships also varied among inference methods in the whole-genome study by Ronco et al. (2021). On the other hand, some nodes with low support showed similar NQS values for only two alternative topologies. For example, in the node supporting the monophyly of LCR *Lamprologus* (NQS t1 = 0.39, t2 = 0.36, t3 = 0.25), the second most common alternative topology resolved ([*L. lethops, L*. sp. “Fingu”], *L. teugelsi*) as the sister lineage to the other CR *Lamprologus*. For the sister relationship of *L. teugelsi* and *L. lethops + L*. sp. “Fingu” (NQS t1 = 0.35, t2 = 0.29, t3 = 0.34), the second most frequent alternative topology supported *L. teugelsi* as sister to the LCR species ([*L. tigripictilis, L. markerti*]*, L. werneri*), and *L. lethops* + *L*. sp. “Fingu” sister to them. Finally, regarding the sister relationship of *L. congoensis* and *L. congoensis* “Kwilu” (NQS t1 = 0.37, t2 = 0.28, t3 = 0.35), the second most common topology resolved *L. congoensis* as the sister lineage to *L. tumbanus* + *L. mocquardi*, with *L. congoensis* “Kwilu” sister to them all (Supplementary Fig. S3). It is important to mention that all AU tests among alternative topologies were not significant, indicating that none of the ML phylogenetic hypotheses proposed for the CR *Lamprologus* are significantly better supported by the data (Supplementary Table S3).

### Network and Hybridization Analysis

The phylogenetic network inferred using SplitsTree (Fig. 3c) showed long edges and high support for most CR *Lamprologus*, suggesting high confidence for their respective monophyly. On the other hand, in agreement with previous results, relationships between taxa displayed a high degree of reticulation, suggesting contradictory evidence for their grouping, for example between subclades of *L. congoensis, L. congoensis* “Kwilu” and *L. mocquardi* “Nki” and *L. mocquardi* “Kisangani”, and between *L. lethops* + *L*. sp. “Fingu” and *L. teugelsi* (Fig. 3c). The average delta score for this data set was 0.27, which suggests that phylogenetic relationships are only moderately tree-like. However, the contribution to deviations from tree-like patterns is not the same across taxa, with *L. lethops* showing the highest taxon-specific score (Delta = 0.336, SD = 0.200) followed by *L. congoensis* (Delta = 0.309, SD = 0.027), *L. congoensis* “Kwilu” (Delta = 0.296, SD = 0.020), and *L. werneri* (Delta = 0.292, SD = 0.028) (Supplementary Fig. S4).

We found many instances of hybridization (|*Z*-scores| > 3 and *P* < 0.01) in 62 and 66 of 120 combinations of species triplets analyzed with the ABBA-BABA test for the MSC and the ML trees, respectively (Supplementary Tables S4 and S5). For *D-* and *f*-statistics, highest values were observed in tests that included *L. congoensis* and *L. werneri* as the P2 or P3 taxa, indicating an excess of shared derived alleles suggesting introgressive hybridization between them.

**Figure 4.**
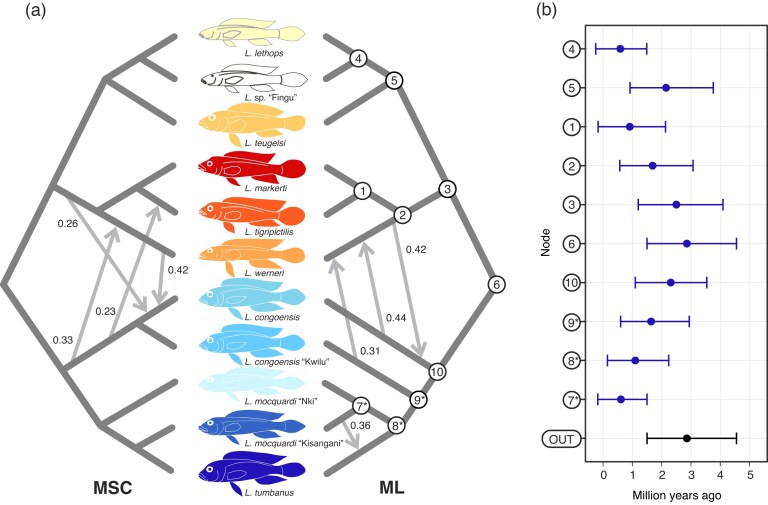
a) Graphical representation of the major hybridization events inferred between species of CR *Lamprologus* based on the two main phylogenomic hypotheses using DSuite (MSC: species tree and ML: maximum likelihood). The numbers next to arrows show the *f*-branch statistic values. b) Estimated ages (Mean tMRCA ± 95% HPD) for the nodes in the CR *Lamprologus* clade based on the fossil-calibrated timetree constructed in BEAST2. The numbers with an asterisk indicate different relationships in the MSC and ML trees. Node OUT represents the tMRCA between the CR *Lamprologus* clade and all LT lamprologines in the outgroup.

Similarly, summary *f*-branch statistics displayed the highest indication of introgression between clades containing *L. congoensis* and *L. werneri*. When considering the MSC species tree topology, the highest *f*-branch values were observed between *L. werneri* and *L. congoensis* (*f_b_*= 0.41) and between the branch leading to *L. congoensis* + *L. congoensis* “Kwilu” and *L. werneri* (*f_b_*= 0.33). When considering the ML tree topology, the highest *f*-branch values were between *L. congoensis* and *L. werneri* (*f_b_* = 0.44), and *L. werneri* and *L. congoensis* (*f_b_* = 0.42) (Fig. 4a). Many other potential instances of introgression were identified, and the majority involved *L. congoensis* (22–24 significant *f*-branch values; Supplementary Figs. 11 and 12 and Supplementary Table S6).

### Phylogenetic Analysis of Mitochondrial DNA

In contrast to our UCE-based analysis, the phylogenetic hypothesis of Lamprologini based on the mitochondrial ND2 gene resolved CR *Lamprologus* into two non-sister clades nested across the LT radiation (Fig. 2b and Supplementary Figs. S1 and S2). One clade, herein the “mainly LCR” mitochondrial clade (BS = 100, PP = 1.0), was resolved as sister to *N*. sp. “palmeri” and *N. gracilis* and related to other LT *Neolamprologus*. All species were reciprocally monophyletic (BS > 87, PP > 0.97), as also reflected in the haplotype network (Supplementary Figs. S5 and S6), but the relationships within and among species lacked strong support. The other clade, herein the “mainly CUCR” mitochondrial clade, was resolved as the sister group to *T. vittatus* and related to other *Telmatochromis* and *Neolamprologus* species. Interestingly, *L. symoensi—*the only riverine *Lamprologus* not included in the UCE phylogenomic study—was found in this group of related species but clearly positioned outside the two mitochondrial clades of CR species (Supplementary Figs. S1 and S2, see Taxonomy and Nomenclature section). This species, known from the Upemba lakes (Democratic Republic of the Congo) in the upper Lualaba River, was recovered alongside the riverine lamprologine *T. devosi*, which was identified as sister group to the *L. symoensi* mitochondrial clade. The clade including *T. devosi* and *T. symoensi* has also been associated with the recently described *T. salzburgeri* from the Lufubu River (Indermaur et al. 2024). Notable patterns in the distribution of species in the two mitochondrial clades include the observations that *L. werneri* is the only LCR species not found in the “mainly LCR” clade and conversely, *L. congoensi*s from Kwilu is not found in the “mainly CUCR” clade.

The age of the MRCA of all the lamprologine ND2 haplotypes was 8.57 Ma (95% HPD 6.07–12.02 Ma) and the MRCA of the two mitochondrial lineages was 8.29 Ma (95% HPD 6.14–11.6). The crown nodes for the “mainly LCR” and “mainly CUCR” clades were estimated at 3.11 Ma (95% HPD 1.94–4.56 Ma) and 2.57 Ma (95% HPD 1.56–3.76 Ma), respectively (Supplementary Fig. S7).

### Population Genomic Analysis

**Figure 5.**
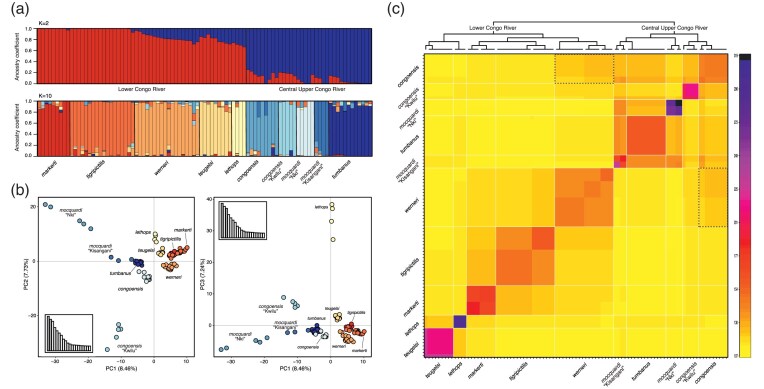
a) Ancestry coefficients of CR *Lamprologus* species as inferred from sNMF for the most conservative (*K* = 2) and best-fitted values of *K* (*K* = 10). Each bar represents an individual sample. b) Scatterplots of the PCA built with one SNP per UCE locus in adegenet. The differences between the central upper Congo and the lower Congo River samples are explained by PC1 (8.46%). PC2 (7.73%) and PC3 (7.24%), respectively, explain genetic differences of *L. congoensis* from Kwilu and *L. lethops* relative to other taxa. c) Co-ancestry matrix obtained in fineRADStructure. The dashed, black box highlights *L. werneri* and *L. congoensis* samples with higher shared co-ancestry between, rather than within regions in the Congo River.

Admixture analysis performed with sNMF confirmed the genetic clustering of each species, although there was evidence of shared alleles across species. We inferred an optimum number of *K* = 10 clusters (Fig. 5a and Supplementary Fig. S8) that corresponded with the clades proposed by the phylogenomic hypotheses and the SplitsTree network (Fig. 3c). When forced to *K* = 2, the two genetic groups corresponded to the LCR and the CUCR species (Fig. 5a and Supplementary Fig. S9).

The main axes of the PCA in combination explained 7.24–8.46% of genetic variation (Fig. 5b). Axis PC1 ordinated samples along a gradient from CUCR to LCR species. Axis PC2 revealed high genetic differentiation of *L. congoensis* “Kwilu” and *L. mocquardi* “Kisangani” relative to all the other species. Similarly, most of the variation explained by axis PC3 was due to the high genetic differentiation of *L. lethops*. Overall, LCR species, except *L. lethops*, were more similar to each other than to CUCR species, which were more dispersed across the ordination space, except for *L. mocquardi* “Nki,” *L. tumbanus*, and *L. congoensis* that tended to cluster together.

The fineRADstructure analysis was consistent with previous results, with each species assigned to distinct genetic clusters sharing higher coancestry levels within, than between them (Fig. 5c). Overall, species clustered in two groups corresponding to the LCR and CUCR species. A notable exception was *L. werneri*, which, despite clustering with LCR species, showed higher levels of coancestry with some species in the CUCR, such as *L. congoensis* (Fig. 5c).

## Discussion

Understanding what mechanisms drive the enormous diversity of tropical freshwater organisms remains a fundamental question in evolutionary biology. The African Great Lakes epitomize the incredible richness of cichlid fish communities, with adaptation and hybridization considered key diversification drivers in these lacustrine systems (Salzburger et al. 2005; Loh et al. 2013; Brawand et al. 2014; Irisarri et al. 2018; Ronco et al. 2021). Nevertheless, certain river basins also harbor a significant portion of the region’s biodiversity, and have been shown to be important sources of allelic diversity shaping adaptation in lake cichlids (Meier et al. 2017). Although vicariance is generally considered the main driver of diversification in riverine systems, recent studies suggest more complex histories involving intricate geological, ecological, and climatic factors (Kurata et al. 2024). These studies remain scarce, and our understanding of the relationships between riverine and lacustrine communities and the similarities and differences in their diversification processes is still incomplete. Our work bridges this knowledge gap and uses UCE and mitochondrial data in conjunction with phylogenomic and population genomic methods, along with the most extensive sampling of riverine lamprologines to date. Our analyses help clarify the origin and diversification patterns of the CR lamprologine radiation despite extensive ILS and introgression in this clade.

### Untangling Phylogenetic Signal, ILS, and Hybridization

Although our phylogenomic analysis supports previous phylogenetic hypotheses about *Lamprologus* from the Congo River, such as their monophyly and a nested origin within the Lake Tanganyika radiation, different inference methods, considered separately, were sometimes inconclusive or contradictory regarding relationships within the CR clade. These discrepancies likely stem from multiple factors, including low or heterogeneous phylogenetic signal, ILS, introgressive hybridization, or systematic errors arising from model misspecification. Such misspecifications can be driven by heterogeneous base composition, variation in substitution rates or branch lengths, and missing data, among other factors (Mirarab et al. 2014; Arcila et al. 2017; Shen et al. 2017; Bravo et al. 2018; Alda et al. 2019; Simon 2022).

UCE loci are short and relatively invariant molecular markers, which can result in individual gene trees with low support, especially in rapid radiations (Faircloth et al. 2012). Their strength lies in the large number of loci that can be sequenced and analyzed (Gilbert et al. 2015). However, in the present study, the average nodal bootstrap value across gene trees was only 11.43 (±17.83 SD), raising the question of whether we should consider conflicting or unsupported relationships as unresolved. AU topology tests based on the concatenated data set and ML tree were non-significant for all alternative hypotheses, suggesting that the data may support them all equally (Supplementary Table S3). However, this test assumes that sequence data are generated from a single tree and does not account for gene tree discordance (Sayyari and Mirarab 2018). On the other hand, a polytomy test based on quartet frequency distributions under the MSC model found that 7 out of the 10 nodes in the CR *Lamprologus* radiation were sufficiently supported by the data to rule out polytomies. However, for three nodes within the radiation, as well as for the relationship between CR *Lamprologus* and their closest LT relative, the polytomy test was non-significant (Fig. 3a).

Inability to reject the null hypothesis could indicate that these are true polytomies or simply may reflect a lack of signal to resolve those relationships (Townsend et al. 2012; Sayyari and Mirarab 2018). Branch lengths and the number of informative gene trees would determine whether the null hypothesis is rejected, and as expected, the shortest branches in our species tree (<0.1 coalescent units) had *P*-values > 0.05 (Fig. 3a and Supplementary Fig. S10), corresponding to relationships with low support or incongruent across data sets and methods.

Although we might expect that nodes for which the null hypothesis is not rejected would contain fewer informative gene trees, their number remained constant across all nodes, indicating that this factor may not be affecting nodal *P*-values or LPP support (Fig. 3b). The recent study of Astudillo-Clavijo et al. (2023), which analyzed many hundreds of exon loci, encountered similar challenges—including low gene tree concordance across all nodes of the CR clade and inconsistencies between inference methods, particularly those involving the relationships of *L. werneri, L. teugelsi*, and *L. lethops*. These results suggest that simply adding more data may not resolve the problem (Philippe et al. 2011). Indeed, we found indirect support for this by recalculating species trees after collapsing poorly supported branches in gene trees at varying thresholds, and we observed that the *P*-values of the polytomy test decreased as the contraction threshold increased (Supplementary Fig. S10). Although this may appear counterintuitive, as reducing the number of effective gene trees for each node could decrease the test’s power, collapsing nodes with low support also reduces noise by retaining only those relationships consistently recovered across gene trees. This contrasting effect demonstrates that the likelihood of resolving certain nodes may increase despite analyzing fewer gene trees; this observation suggests it is not a lack of signal but a result of noise reduction that favors resolution. Overall, this result aligns with similar scenarios where, at short time scales, random heterogeneity from ILS or deviations from the MSC model, rather than low informativeness and systematic error, cause phylogenetic discordance (Sayyari and Mirarab 2016; Alda et al. 2021).

High levels of gene tree heterogeneity across the tree of Lamprologini are also reflected in the low NQS values obtained, indicating that on average less than half of observed quartets in the input gene trees are also recovered in the species tree (mean NQS = 0.455 ± 0.108). We can attribute a large portion of this heterogeneity to ILS, given that at least 10 out of 58 nodes in the species tree showed virtually the same NQS (∼33.33%) across all possible quartets. These findings match our expectations and previous observations in the EAR adaptive radiation, in which the rapid formation of lineages has facilitated the retention of ancestral polymorphisms between species (Koblmüller et al. 2010; Brawand et al. 2014; Meyer et al. 2015, 2017; Weiss et al. 2015). Clearly, ILS is not exclusive to lacustrine species but may also be pervasive in rapid riverine radiations (Schwarzer et al. 2011, 2012a; Loh et al. 2013; Alter et al. 2017; Astudillo-Clavijo et al. 2023).

Deep coalescence may be hindering our ability to resolve the relationships of the blind cichlid, *L. lethops*. Although both phylogenomic and population genomic analyses agreed on its affinity with *L*. sp. “Fingu” and *L. teugelsi*, and to this lineage being the sister group to the remaining LCR species, this result was weakly supported and, based on the polytomy tests, we could not reject that these relationships are real polytomies (Figs. 2a and 3a,b). Our results using UCE data parallel those obtained using exons, where relationships of *L. lethops* varied depending on the analysis conditions and showed virtually equal quartet support for all possible topologies. Remarkably, only 4.93% of the exon gene trees contained a branch supporting *L. lethops* + *L. teugelsi* as the sister lineage of all LCR species (Astudillo-Clavijo et al. 2023; N.B.: these authors did not have access to *L*. sp. “Fingu”), suggesting that strong ILS may result from fast divergence and speciation predating allele fixation. Other processes, probably in combination with ILS, may be further hindering phylogenetic resolution such as population bottlenecks and isolation that has resulted in accelerated genetic drift, low diversity, and high genotypic and phenotypic divergence (Aardema et al. 2020; Stiassny and Alter 2021; Kurata et al. 2022, 2024). For example, the SplitsTree network showed the longest branches and the highest delta scores for *L. lethops + L*. sp. “Fingu,” indicating high differentiation and gene tree dissimilarity for the splits involving these species (Supplementary Fig. S4). Although elevated delta scores can suggest hybridization, our ABBA-BABA analysis did not detect signals of introgression in this case. However, simulation studies have demonstrated that very weak introgression can result in low or non-significant *D*-statistics. Additionally, *D*-statistics can be affected by homoplasy and reversals, which tend to accumulate along long branches in a phylogeny (Koppetsch et al. 2024), such as those observed in *L. lethops*.

Unresolved or inconsistent relationships may also reflect the differing efficacy of phylogenomic methods to accommodate gene tree heterogeneity (Degnan and Rosenberg 2009; Mirarab et al. 2014; Bravo et al. 2018; Alda et al. 2019). Under these conditions, we may assume that the MSC species tree is more accurate than concatenation-based analyses that can be statistically inconsistent under the MSC and result in incorrect trees despite high support (Kubatko and Degnan 2007; Mirarab and Warnow 2015; Roch and Steel 2015). However, MSC-consistent methods may also reconstruct an erroneous species tree when there is gene flow between species because this violates the assumption that ILS is the sole source of gene tree conflict (Solís-Lemus et al. 2016). In the exon-based phylogenomic study of Astudillo-Clavijo et al. (2023), ILS was considered the most common source of disagreement across the African cichlid tree, although they did not discount hybridization or gene tree error could also affect their inferences. In fact, and as in previous studies (Salzburger et al. 2005; Koblmüller et al. 2010; Meyer et al. 2015, 2017; Weiss et al. 2015; Irisarri et al. 2018), they found that hybridization is widespread in the EAR clade, including among lamprologines. However, within the CR *Lamprologus* clade, they only detected a single instance of possible introgression.

In the present study, by combining nuclear and mitochondrial DNA evidence from multiple samples per species, we were able to decipher a more complex history of hybridization, isolation, and introgression following secondary contact. First, the contrasting non-monophyly of mitochondrial haplotypes and their older age than for nuclear lineages supports the “melting pot Tanganyika” hypothesis, where independent, ancient (riverine and lake), and more recent (lake) lineages amalgamated into the current incarnation of Lake Tanganyika (Weiss et al. 2015). This pattern could be compatible with a hybrid origin of the CR *Lamprologus* that resulted from this fusion. However, given the non-recombining nature of mitochondria, it is unlikely that a hybrid swarm colonized the Congo River while still producing the observed geographical structure of haplotypes. Alternatively, this result suggests that hybridization between ancestral mitochondrial lineages may have occurred outside of Lake Tanganyika.

If the ancestor of the riverine clade originated in Lake Tanganyika and later colonized the Congo River, the two mitochondrial lineages may have persisted as ancestral polymorphisms, co-occurring before sorting into the lower and upper portions of the Congo River. The sorting of these lineages may have been a result of vicariance. This scenario does not exclude the possibility of differential selection acting against mitochondrial haplotypes in either region (Hulsey et al. 2016), which could have further reinforced their segregation. Nevertheless, we consider independent colonization of the Congo River by the two mitochondrial lineages, followed by later hybridization, to be the more plausible explanation.

Other clades of cichlids (*Nanochromis, Steatocranus*, and *Teleogramma*) and spiny eels (*Mastacembelus*) have also colonized the Congo River and the LCR multiple times in the last ∼1–8 myr (Schwarzer et al. 2011; Alter et al. 2015, 2017), which is congruent with the minimum age of dispersal of *Lamprologus* defined by our estimates of the MRCA between the Congo River and the LT lamprologines (95% HPD 3.07–6.23 Ma, Supplementary Fig. S7). Regardless of the temporal sequence of dispersal from LT, the ancestral mitochondrial lineages may have occupied different areas of the proto-Congo River, coming into contact and hybridizing. Our results locate the contact zone between the two lineages in the region of Pool Malebo (Fig. 1) and suggest that introgression may have been restricted by the developing geographical barriers in the LCR (see below) that today limit gene flow (Markert et al. 2010; Schwarzer et al. 2011; Alter et al. 2017; Kurata et al. 2022). Additionally, differences in effective population sizes, cytonuclear incompatibilities, or asymmetric behavioral reproductive isolation (Nevado et al. 2011; Willis et al. 2014) may have influenced this introgression. For instance, we found higher mitochondrial introgression from the CUCR into the LCR clade than vice versa, suggesting that gene flow occurred preferentially from upstream populations. Furthermore, we uncovered two instances of complete mitochondrial capture in opposite directions, indicating that introgression can occur bidirectionally.

We conjecture that newly generated genetic variation may have facilitated the initial diversification of the CR clade, as observed in other cichlid radiations (Meier et al. 2017, 2023; Irisarri et al. 2018), and that subsequent speciation may have been influenced by additional hybridization events (Koblmüller et al. 2007). The combined ABBA–BABA and timetree analyses detected signals of nuclear introgression, which may have occurred along a branch that could date back as far as ∼3.5 Ma. Given that *f*_4_-ratios between multiple taxa are interdependent due to shared genetic drift (i.e., they share branches on the phylogeny), the observed correlations between *f*_4_-ratio scores allowed us to infer that gene flow likely occurred along this branch, as high scores were consistently shared across all of its descendant lineages (Supplementary Figs. S11 and S12, Tables S4–S6). More recent introgression events cannot be completely ruled out, but a single ancestral introgression event could also explain multiple elevated *f_4_*-ratios that have been detected (Malinsky et al. 2021).

This period coincides with the early stages of divergence of the LCR and CUCR clades and predates subsequent microallopatric radiations, such as the *tigripictilis–markerti*–*werneri* species group (Fig. 4 and Supplementary Fig. S7). Interestingly, most gene flow events were inferred between branches of the LCR and the CUCR clades rather than within them, with the strongest signals involving the branches leading to *L. tigripictilis, L. markerti*, and *L. werneri*, and *L. congoensis*, and *L. congoensis* “Kwilu” (Supplementary Figs. S11 and S12, Tables S4–S6). Specifically, *L. werneri* and *L. congoensis* showed the highest fractions of introgressed genomes and higher shared co-ancestry between them than with species of the same clade (*f*-branch statistics = 0.31–0.44; Figs. 4a and 5c). Gene flow between these taxa was also detected using exon data, indicating a relatively high proportion of genes transferred during hybridization (γ = 0.23; Astudillo-Clavijo et al. 2023). This pattern of gene flow between specific LCR and CUCR branches is more consistent with hybridization following independent colonization of the CR rather than a hybrid swarm scenario, the latter of which predicts nuclear introgression at the base of the radiation (Svardal et al. 2021).

Despite the nuclear data detecting gene flow in both directions, the signal from mitochondrial DNA only showed introgression into *L. werneri*, which, furthermore, is the only LCR species that has undergone a complete mitochondrial DNA capture by the CUCR mitochondrial lineage. Similarly, gene flow detected between *L. congoensis* and *L. tigripictilis* is also congruent with their mitochondrial signal because all *L. tigripictilis* collected at Mbelo show CUCR mitochondrial haplotypes that are shared (or are very similar) with *L. werneri* and *L. congoensis*. On the other hand, the downstream populations of *L. tigripictilis*, from which they are genetically differentiated (Figs. 2a and 5c), show unique and divergent LCR mitochondrial haplotypes (Fig. 2b and Supplementary Figs. S5 and S6). Based on these patterns, we argue that introgression from *L. congoensis* must be younger than ∼1.8 Ma—probably after the divergence of the *L. tigripictilis* populations—and geographically limited to the northern portion of the LCR, given that *L. markerti*, despite being more closely related to *L. tigripictilis* than *L. werneri*, shows no signal of mitochondrial or nuclear introgression.

Gene flow between clades of CR *Lamprologus* is also evident in another instance of mitochondrial capture in *L. congoensis* from the Kwilu River, which drains into the Kwa, ∼130 km upstream of Pool Malebo (Fig. 1a). Unlike previous hybridization scenarios, *L. congoensis* “Kwilu” is the only CUCR taxon with LCR haplotypes. The introgressed mitochondrial haplotypes form a monophyletic and distinct group, suggesting a more ancient introgression (Fig. 4a), which may have resulted from an ancestral or extinct lineage (i.e., mitochondrial fossil; Bossu and Near 2009), or the isolation of the Kwilu and the Congo River populations due to drainage reconfiguration that promoted their genetic divergence.

As observed in numerous other cichlid clades, the highly reticulated evolutionary history of CR *Lamprologus* (Fig. 3c) may contribute to the difficulty in resolving their phylogenetic relationships (Solís-Lemus et al. 2016). For example, the contrasting placements of *L. congoensis* and *L. mocquardi*—where *L. congoensis* is monophyletic, and *L. mocquardi* is paraphyletic in the MSC species tree, but the reverse is found in the ML tree (Figs. 2a and 3a)—may reflect biases in the inference methods due to hybridization. First, evaluation of the NQS of nodes involving these taxa indicated that the two quartets corresponding to the MSC and ML topologies have frequencies >33%, suggesting that gene tree discordance is not random or only due to ILS (Fig. 3b). Second, the hybridization test, when carried out assuming the ML tree topology, inferred additional hybridization events between *L. mocquardi* “Kisangani” and *L. tumbanus* that indicate an excess of shared alleles between these non-sister species and are therefore necessary to reconcile the monophyly of *L. mocquardi* hypothesized using concatenation.

### Diversification in the Congo River

Our proposed ages for the origin and diversification of CR *Lamprologus* align well with previous studies, and the geological history of the region. We dated the MRCA of the CR clade to approximately 3.7 Ma (95% HPD 2.5–5.55 Ma), and its split from LT relatives at 4.28 Ma (95% HPD 3.07–6.23 Ma). Our estimate for the MRCA of CR *Lamprologus* and LT lamprologines is roughly 1 myr older than that proposed by Ronco et al. (2021) based on whole-genome data, although the 95% HPD intervals overlap. This discrepancy could reflect differences in data set composition and taxon sampling—specifically, their inclusion of nearly all LT endemics but only one representative of the CR lineage. These times are also consistent with ages proposed for other Congo River cichlids and coincide with the Miocene-Pliocene transition, a period during which the Congo River likely acquired its current configuration (Stankiewicz and de Wit 2006; Runge 2008; Schwarzer et al. 2011; Alter et al. 2017; Stiassny and Alter 2021).

The split times of the two mitochondrial lineages from their closest lacustrine relatives occurred in a similar timeframe (MRCA “mainly CUCR” lineage and rest of LT: 3.53 Ma; 95% HPD 2.31–5.09 Ma, and MRCA “mainly LCR” lineage and rest of LT: 4.18 Ma; 95% HPD 2.63–6.18 Ma; Supplementary Fig. S13) matching previous mitochondrial studies (Schedel et al. 2019). However, when comparing these results, we must first consider that the nuclear estimate is more correlated with the timing of hybridization between the parental lineages that seeded the radiation rather than their dispersal from LT and colonization of the Congo River. Secondly, the estimates are influenced by the methods used and the inherent differences in coalescent times of mitochondrial and nuclear genomes (Neigel and Avise 1986; Hudson 1990). These differences may be further amplified by the extensive reticulation in our data set, leading to alleles coalescing deeper in time (Maddison 1997; Sang and Zhong 2000). Indeed, our UCE-based estimates are older than those obtained with other nuclear markers such as AFLPs or RADs (Sturmbauer et al. 2010; Kurata et al. 2024). Nonetheless, our age estimates indicate that dispersal, colonization, and hybridization events all occurred within a relatively short timeframe. Both ancestral lineages may have dispersed via the Lukuga River that first connected Lake Tanganyika and the Congo River 1.1–3.5 Ma (Lezzar et al. 1996; Cohen et al. 1997), and it seems that the ancestor of the “mainly LCR” mitochondrial lineage entered the Congo River slightly earlier than the “mainly CUCR,” which could have allowed the former to disperse further west to occupy the lower portion of the (proto-) Congo River. A similar hypothesis has been proposed for *Nanochromis* and *Steatocranus*, both of which have undergone two separate colonizations of the Congo River where the younger species occurs in the upper and middle LCR (Schwarzer et al. 2011).

The formation of the LCR rapids likely played a key role in creating barriers to gene flow in CR *Lamprologus* (Kurata et al. 2022, 2024). The estimated split time between the two clades of CR *Lamprologus* (3.7 Ma; 95% HPD: 2.5–5.5 Ma) and their distribution and contact zone around Pool Malebo are fully congruent with geological evidence, indicating that during the Pliocene (ca. 5 Ma), tectonic activity and river captures caused the Palaeo-lake in the Cuvette Centrale to spill over a rocky sill at the start of the LCR at Pool Malebo, creating a new outlet for the Congo drainage (Runge 2008) and shaping biogeographic barriers for fishes (Markert et al. 2010; Lowenstein et al. 2011; Alter et al. 2015). Then, speciation within each clade at either side of the barrier rapidly followed in a 2 myr-timespan characterized by interspersed periods of isolation and connectivity.

Geographical isolation and geographical distance have significantly shaped the processes of speciation and hybridization in the Congo River (Markert et al. 2010; Schwarzer et al. 2011, 2012a; Alter et al. 2017; Kurata et al. 2022, 2024). Yet, the relative importance of these factors may differ depending on the region. For example, in the central and upper sections, the Congo River meanders over sandy substrates with relatively constant water flow, so river network configuration and isolation by distance may be more important drivers of species divergence (Thomaz et al. 2016), given the limited dispersal capabilities of these species (Stiassny and Alter 2021; Kurata et al. 2022). In contrast, in the LCR, cichlid diversification has been mainly attributed to vicariant processes that are a consequence of the heterogeneous landscape, rocky substrate, high-energy rapids, and extreme bathymetric changes (Markert et al. 2010; Schwarzer et al. 2011; Alter et al. 2017; Stiassny and Alter 2021; Kurata et al. 2022, 2024). Some studies have also invoked *in situ* adaptive processes to link these radiations to the emergence of new habitats in the LCR (Schwarzer et al. 2011; Alter et al. 2015). Additionally, hybridization may have facilitated these adaptations by increasing heritable genetic variation and, depending on the context, enabling populations to explore new fitness peaks or even generate novel phenotypes (Seehausen 2004; Schwarzer et al. 2012a; Svardal et al. 2021). The most striking consequence of these evolutionary processes is the cryptophthalmic cichlid *L. lethops*. Despite the high phylogenomic uncertainty about the relationships of this species (Figs. 2a and 3; Schedel et al. 2019; Astudillo-Clavijo et al. 2023; Jimenez et al. 2024), our combined evidence supports its long-term isolation, reveals cryptic diversity that warrants further exploration (e.g., *L*. sp. “Fingu,” see Taxonomy and Nomenclature section), and indicates a closer evolutionary relationship with the southern rapids samples of *L. teugelsi* than with species from the middle or northern rapids. Interestingly, this pattern mirrors the microallopatric pattern observed in the other clade of LCR *Lamprologus*, ([*L. tigripictilis, L. werneri*] *L. markerti*), and the co-occurring *Nanochromis* and *Steatocranus*, in all of which the Inga rapids have played a vicariant role (Markert et al. 2010; Schwarzer et al. 2011; Kurata et al. 2022).

To explain the extensive reticulation of the CR *Lamprologus* tree, populations from both regions of the Congo River must have been in contact at different times. Based on the *f*-branch statistics, hybridization across species groups may have occurred as early as 2.58 Ma (95% HPD: 1.57–4.07 Ma) before the divergence of *L. tigripictilis, L. markerti, L. werneri*, and until 1.84 Ma (95% HPD: 0.83–3.13 Ma) (Fig. 4). Although we found evidence for many possible cases of hybridization, some of these inferences may be inflated because a single gene flow event can result in multiple significant *D*- and *f*-statistics (Malinsky et al. 2021). It is possible that the multiple water level fluctuations during the late Pliocene and Pleistocene both isolated and reconnected populations during periods of low and high water discharge, respectively (deMenocal 2004; Takemoto et al. 2015; Stiassny and Alter 2021; Kurata et al. 2024). For instance, corridors along riverbanks during wet periods, such as interglacials, could explain secondary contacts between populations such as *L. werneri* in the main channel of the Congo River and *L. congoensis* in the Kwilu River. Additional sporadic events like tectonic activity could also explain these connections (Thomaz et al. 2016).

### Taxonomic and Nomenclatural Implications

**Figure 6.**
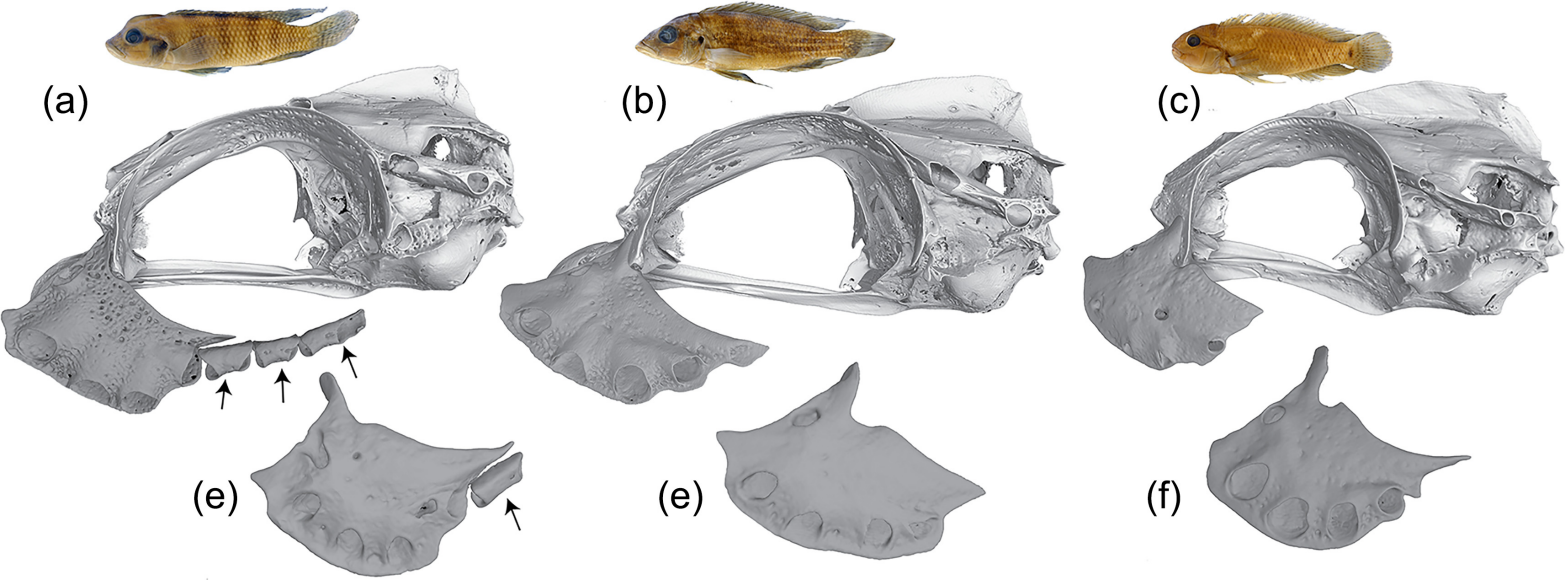
Infraorbital series *in situ* in a) *Lamprologus congoensis* (type species), b) *Neolamprologus tetracanthus* (type species), and c) *Telmatochromis temporalis* (type species). Isolated series in d) *Lamprologus symoensi*, e) *Neolamprologus mondabu*, and f) *Telmatochromis devosi*. The arrows indicate derived presence of 1–3 infraorbital elements adjacent to first infraorbital (lachrymal).

Ronco et al. (2020) described LT Lamprologini diversity as comprised of *Altolamprologus* (2 spp.), *Chalinochromis* (3 spp.), *Julidochromis* (6 spp.), *Lamprologus* (10 spp.), *Lepidiolamprologus* (7 spp., *L. cunningtoni* assigned in error), *Neolamprologus* (51 spp., including *N. cunningtoni*), *Telmatochromis* (6 spp.), and *Variabilichromis* (1 sp.). Although sampling for this study is focused on Congo River species and coverage for Lake Tanganyika taxa is just 50%, our results, although differing in many details, broadly accord with those of Day et al. (2007; 79% LT coverage), Sturmbauer et al. (2010; 88% coverage), and Ronco et al. (2021; 96.6% coverage). As noted by those authors there is a striking lack of concordance between current generic assignments and phylogenetic placements, most notably among the numerous *Neolamprologus* and *Lamprologus* species arrayed across both the “ossified” and a “non-ossified” clade. Full nomenclatural resolution for this taxonomic mismatch requires detailed revisional work and is clearly beyond the scope of the present study. However, in light of the recognition of a well-supported CR clade, composed entirely of *Lamprologus* species and including the type species of the genus, *Lamprologus congoensis* Schilthuis 1891, we propose the restriction of *Lamprologus* to Congo River taxa only (Supplementary Table S7). Morphological support for this definition is found in the shared possession of a derived configuration of the infraorbital series. As noted by Stiassny (1997) and Schelly and Stiassny (2004) all CR *Lamprologus* possess robust, well-developed infraorbital elements (1–3, usually 2) adjacent to the first infraorbital (lachrymal), and these are primitively lacking in LT lamprologines and in the riverine species, *T. devosi* (Fig. 6). To accommodate the former LT *Lamprologus* species, and as a temporary expedient, we propose their generic reassignment to *Neolamprologus* Colombé and Allgayer 1985. Although we recognize that this does little to ameliorate the nomenclatural problems, and ultimately the generic assignments necessary for the diverse species of LT *Neolamprologus*, it does at least render *Lamprologus* monophyletic. A new listing of all described species to be included in *Neolamprologus* is provided (Supplementary Table S7).

**Figure 7.**
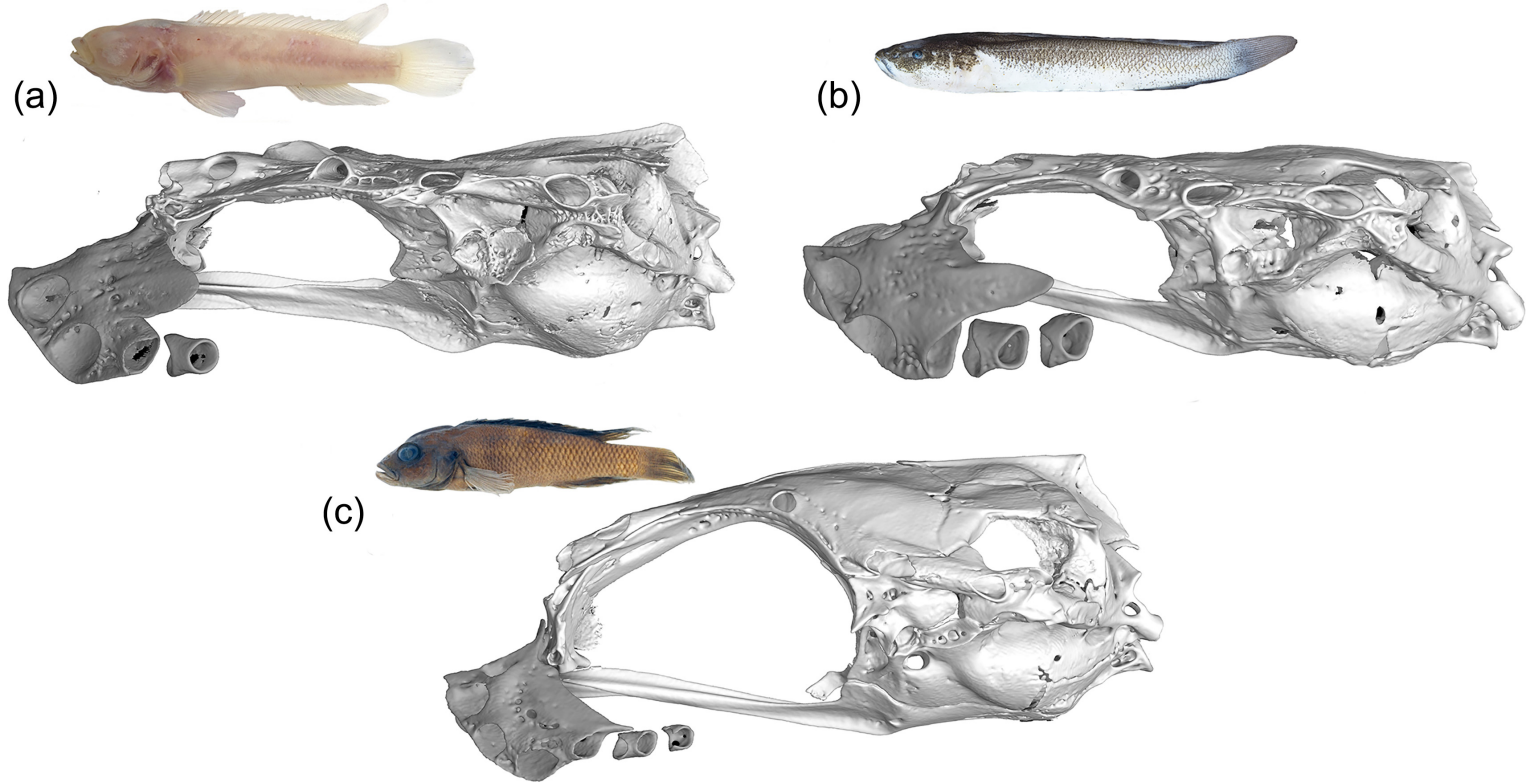
Lateral view of neurocranium with *in situ* infraorbital elements in a) *Lamprologus lethops*, b) *Lamprologus* sp. “Fingu,” and c) *Lamprologus teugelsi*.

Despite high levels of ILS and introgression confounding the tree-like resolution of relationships among CR *Lamprologus*, in the main, our phylogenomic data support much of the current taxonomy for morphologically determined species of CR *Lamprologus*. In addition, we show here for the first time the unambiguous resolution of sister relationship of *L. lethops* (Fig. 7a) to L. sp. “Fingu” (Fig. 7b), a species with which it shares many derived morphological features (Stiassny and Alter in preparation). Although their relationship to *L. teugelsi* (Fig. 7c) is less certain, the identity of all sample specimens of *L. teugelsi* is confirmed. However, for some currently recognized taxa our study indicates the existence of novel molecular lineages. These include a grouping of all sampled individuals from the Kwilu River (*L. congoensis* “Kwilu”), which we recover as genetically distinct from other individuals of *L. congoensis* (including specimens from the type locality in the vicinity of Pool Malebo). Similarly, *L. mocquardi* is resolved into two genetically distinct lineages: *L. mocquardi* “Kisangani” and *L. mocquardi* “Nki.” Unfortunately, at the time of our study we were unable to include samples of *L. mocquardi* from the type locality in the Ubangi basin. These have since been obtained and, as for *L. congoensis*, detailed analyses are currently underway to investigate whether morphological differentiation matches that revealed by phylogenomic data.

## Conclusions

The data and analyses presented here demonstrate both the strengths and some of the limitations of current phylogenomic methods in radiations defined by recent divergences, ILS, and/or introgression, underscoring the importance of integrating diverse approaches (e.g., phylogenomic, population genomic, model- and non-model-based) that can accommodate different processes causing discordance. By combining the most comprehensive sample of riverine lamprologines to date with phylogenomic and population genomic analyses of UCE and mitochondrial data, we were able to resolve several outstanding questions related to the diversification of this clade, uncover previously undescribed diversity, and render *Lamprologus* monophyletic by restricting the genus to include only CR species. Our results support extensive hybridization and confirm the monophyly of *Lamprologus sensu stricto*. We reconstructed this clade’s phylogenetic relationships and population structure in the context of biogeographic distributions and identified the sister clade of the enigmatic blind cichlid, *L. lethops*. We estimated divergence times between major clades and these results were congruent with previous studies and with our current understanding of the geological and hydrological history of the region separating CUCR basin species and species endemic to the LCR (defined as downstream from Pool Malebo). Our results suggest that the rapid speciation of *Lamprologus sensu stricto* has been marked by repeated allopatric divergence, secondary contact, and reticulation events that highlight the role of the Congo basin’s complex landscape and highly dynamic hydrology as a diversity pump, fueling diversification within this unique aquatic ecosystem.

## Supplementary Material

syaf032_Supplemental_Files

## Data Availability

Raw read data are archived in the NCBI Sequence Read Archive (SRA; BioProject: PRJNA1097814), and data files (concatenated and individual gene alignments) and supplementary appendices can be found in the Dryad data repository: https://doi.org/10.5061/dryad.8931zcs13.
