# Supplementary material for: Phylogenomic and Population Genomic Analyses of Ultraconserved Elements Reveal Deep Coalescence and Introgression Shaped Diversification Patterns in Lamprologine Cichlids of the Congo River
Source: Syst Biol. 2025 May 13;74(4):600–21. doi: 10.1093/sysbio/syaf032 (PMC12640084; doi:10.1093/sysbio/syaf032)
Supplement: syaf032_Supplemental_Files [file syaf032_supplemental_files.zip › Supplementary_Materials_Appendices.docx]

**Phylogenomic and Population Genomic Analyses of Ultraconserved Elements Reveal Deep Coalescence and Introgression Shaped Diversification Patterns in Lamprologine Cichlids of the Congo River**

Fernando Alda^1, 2, *^, S. Elizabeth Alter^3, 4^, Naoko P. Kurata^3, 5^, Prosanta Chakrabarty^2, 3^, Melanie L. J. Stiassny^3, 6, *^

^1^*Instituto de Investigación en Recursos Cinegéticos (IREC; CSIC-UCLM-JCCM), Ciudad Real, Spain*

^2^*Museum of Natural Science and Department of Biological Sciences, Louisiana State University, Baton Rouge, Louisiana, USA*

^3^*Department of Ichthyology, American Museum of Natural History, New York, New York, USA*

^4^*Department of Biology and Chemistry, California State University Monterey Bay, Seaside, California, USA*

*^5^Department of Natural Resources and the Environment, Cornell University, Ithaca, New York, USA*

^6^*The Sackler Institute for Comparative Genomics, American Museum of Natural History, New York, New York, USA*

Supplementary material

**Supplementary Materials Appendix A:** Laboratory Methods and Bioinformatic Pipelines

**Figure S1.** Maximum Likelihood phylogenetic hypotheses of Lamprologini based on mitochondrial gene ND2 sequences using IQ-TREE2.

**Figure S2.** Bayesian Inference phylogenetic hypotheses of Lamprologini based on mitochondrial gene ND2 sequences using MrBayes.

**Figure S3.** Local posterior probability, normalized quartet score, and number of informative gene trees for the main and alternative quartet topologies for all the nodes in the Congo River *Lamprologus* clade as recovered in ASTRAL-III. N1-N10 correspond to the nodes in the small inset tree at the top.

**Figure S4.** Average Delta scores ± SD for all the species of riverine *Lamprologus* included in the SplitsTree analysis.

**Figure S5.** Haplotype network of the “mainly LCR” clade samples of riverine *Lamprologus* based on mitochondrial gene ND2 sequences using PopArt.

**Figure S6.** Haplotype network of the “mainly CUCR” clade samples of riverine *Lamprologus* based on mitochondrial gene ND2 sequences using PopArt.

**Figure S7.** Calibrated timetree of Lamprologini based on the fixed ML topology using BEAST2. Values indicate mean age estimates and bars represent 95% HPD intervals for each node. The estimates are the combined result of four independent analyses of ~100 UCE loci ran by duplicate.

**Figure S8. Cross-entropy values** as inferred from sNMF to choose the best-fitting (lowest) *K* value for our data values of *K*.

**Figure S9.** Ancestry coefficients of riverine *Lamprologus* species as inferred from sNMF for the most frequent structure arrangements recovered from *K* = 2 through K = 10. Each bar represents an individual sample.

**Figure S10.** Polytomy test results for three datasets using all available gene trees with nodes collapsed at increasing thresholds of bootstrap support (BS) a) all nodes included, b) nodes with BS>1 included, c) nodes with BS>10 included. In the x-axis we show, for each internal branch in the ASTRAL species tree, its estimated length in coalescent units (CU) in log scale, and in the y-axis the polytomy test 1-*p*-value and the local posterior probability (LPP) estimated in the species tree. The dashed line indicates the significance level (1-*p*-value = 0.95) of the polytomy test.

**Figure S11.** *f*-branch test results for the ASTRAL tree topology.

**Figure S12.** *f*-branch test results for the ML tree topology.

**Figure S13.** Calibrated timetree of mitochondrial ND2 gene sequences of Lamprologini using BEAST2. Values indicate mean age estimates and bars represent 95% HPD intervals for each node. Samples in red represent the “mainly LCR” clade and samples in blue represent the “mainly CUCR” clade.

**Supplementary Materials Appendix A: Laboratory Methods and Bioinformatic Pipelines**

*Library Preparation*

We extracted total DNA from 159 tissue samples (Table S1a) using Qiagen DNeasy Tissue kits following manufacturer’s instructions. We randomly sheared 100-500 ng DNA by sonication to a target size of 400-600 bp using a EpiSonic Multi-Functional Bioprocessor (Epigentek) and used it to construct DNA libraries using the Kapa Hyper Prep Kit (Kapa Biosystems). In the ligation step, instead of the standard Illumina adapters, we used adapters containing a custom index sequence (Faircloth and Glenn 2012), and we used a generic SPRI substitute (Rohland and Reich 2012) for all cleanup steps involving magnetic beads. We performed two cleanup steps of the ligation reaction, and quantified 2 µl of the resulting library using a Qubit fluorometer (Life Technologies). We amplified 15 µl of the library volume using a reaction mix of 25 µl HiFi HotStart ReadyMix (Kapa Biosystems), 5 µl of Illumina TruSeq primer mix (5 µM each), and 5 µl ddH_2_O and the following thermal profile: 98 °C for 45 s, 12 cycles at 98 °C for 15 s, 60 °C for 30 s, 72 °C for 60 s; and a final extension of 72 °C for 5 m. We purified completed PCR reactions using 1X SPRI, and resuspended libraries in 33 µl ddH_2_O, and we quantified 2 µl of each library using a Qubit fluorometer. We combined groups of eight libraries at equimolar concentrations, having a final concentration of each enrichment pool of 147 ng/µl in 3.4 µl (500 ng total).

*Target Enrichment, and Sequencing of UCEs*

We enriched libraries using a set of 2001 probes (Actinopts-UCE-0.5Kv1) targeting 500 UCE loci across Actinopterygii (Faircloth et al. 2013). We followed library enrichment procedures for the MYcroarray MYBaits kit v.3.0, except we added 500 ng custom blocking oligos designed against our custom sequence tags, and using 10 inosines to block the 10 nucleotide index sequence. We ran the hybridization reaction for 24 hours at 65 °C. Following hybridization we bound all pools to streptavidin beads (MyOne C1, Life Technologies) and washed bound libraries according to a standard target enrichment protocol (Blumenstiel et al. 2010). We added 30 µl of ddH_2_O to each sample, and combined 15 µl of streptavidin bead-bound, enriched library in ddH_2_O with 25 µl HiFi HotStart Taq (Kapa Biosystems), 5 µl of Illumina TruSeq primer mix (5 µM each), and 5 µl of ddH_2_O. We PCR-recovered each library using the following thermal profile: 98 °C for 45s; 16 cycles of 98 °C for 15s, 60 °C for 30s, 72 °C for 60s; and a final extension of 72 °C for 5m. We placed the resulting PCR reactions in a magnet stand and removed the supernatant to separate the PCR-recovered, enriched DNA (supernatant) from the streptavidin beads. We subsequently purified the enriched DNA for each pool using 1X SPRI, and we re-hydrated enriched DNA in 33 µl ddH_2_O. We quantified 2 µl of each enriched pool using a Qubit fluorometer, and we diluted enriched pools to 2.5 ng/µl in 10 mM Tris-HCl. We combined enriched pools of 8 samples to create an equimolar pool-of-pooled-libraries at 10 nM concentration that we in one lane of PE150 sequencing on an Illumina HiSeq 3000 (Oklahoma Medical Research Foundation).

*Processing of Captured Sequence Data*

We preprocessed demultiplexed sequences and prepared them for analyses using programs in the PHYLUCE package (Faircloth 2016), available at <http://github.com/faircloth-lab/phyluce>. First, we used the program *illumiprocessor* (Faircloth 2013) that uses *trimmomatic* (Bolger et al. 2014) to trim reads and remove adapter contamination and low quality bases, then we assembled cleaned reads using a parallel wrapper around *trinity* (trinityrnaseq-r2013-02-25; Grabherr et al., 2011; Marçais and Kingsford, 2011).

To identify assembled contigs representing enriched UCE loci, we aligned species-specific contig assemblies to a FASTA file of all enrichment baits using *phyluce_assembly_match_contigs_to_probes.py*. This program implements the matching process using LASTZ and ensures that matches are 80% identical over 80% of their length. This program also screens and removes apparent duplicate contigs or contigs that are hit by baits targeting more than one UCE locus.

In addition to the 159 enriched taxa from which we collected new data, we extracted the same UCEs *in silico* for three genome-enabled species *Neolamprologus brichardi* (PRJNA60365) (Brawand et al. 2014), *L. lethops* (n=5) and *L. tigripictilis* (n=1), and one unidentified species *L.* sp. (n=1) (Kurata et al., 2022; Supplementary Table 1) using scripts available at <https://github.com/carloliveros/uce-scripts>. Briefly, genome assemblies were downloaded from online repositories in FASTA format and converted to 2bit format using the faToTwoBit program. We aligned the probe sequences to the genomes using LASTZ (available at http://www.bx.psu.edu/miller_lab), and used the resulting match coordinates map to slice the FASTA files from each respective genome at ±1000 bp of the UCE core matched by the probes using *phyluce_probe_slice_sequence_from_genomes.py*. We then re-assembled sequences and removed contigs that were hit by probes targeting different UCE loci using *phyluce_assembly_match_contigs_to_probes.py*.

We created a list with the names of the enriched and *in silico* UCE contigs and used it as input in the program *phyluce_assembly_get_match_counts.py* to query the relational database created in the previous steps to generate a list containing all loci having data for any taxon. We used these lists to create separate monolithic FASTA files matching each locus list in *phyluce_assembly_get_fastas_froms_match_counts.py*. We exploded each monolithic FASTA by locus and aligned sequence data for loci containing more than four taxa using *phyluce_align_seqcap_align.py* and MAFFT (Katoh and Standley 2013). Following alignment, we used Gblocks 0.91b (Castresana 2000) in *phyluce_align_get_gblocks_trimmed_alignments_from_untrimmed.py* (trimming parameters --b1=0.5; --b2=0.5; --b3=10; --b4=5) to trim and remove ambiguous or poorly aligned portions of the alignments. We removed the locus names from all alignments using *phyluce_align_remove_locus_name_from_nexus_lines.py*.

After screening and removing non-target and duplicated or misassembled contigs, the program creates a relational database containing several tables that map the contig names generated by the assembler to the names of each corresponding UCE locus across all taxa. We used the program *phyluce_assembly_get_match_counts.py* to query the relational database and generate a list containing all loci having data for any taxon. We input this list of loci to an additional program (*phyluce_assembly_get_fastas_from_match_counts.py*) to create separate monolithic FASTA files. We exploded each monolithic FASTA by locus and we aligned sequence data for loci containing more than four taxa using *phyluce_align_seqcap_align.py* and MAFFT.

We filtered the entire set of aligned loci to create an incomplete matrix containing data for at least 75% of the samples (alignments contained ≥ 125 of 167 samples), and computed alignment statistics and the number of informative sites across alignments, and concatenated them into a PHYLIP supermatrix.

*Extraction of SNPs from UCE Data*

We extracted SNPs from UCE data of 93 riverine *Lamprologus* to analyze the diversity, and structure of the Congo River species from a population genomic perspective (full details about the bioinformatic pipeline are provided in Supplementary Materials Appendix A). To identify SNPs and indels, we created a reference dictionary for the complete genome of *L. lethops* using the Picard toolkit (https://broadinstitute.github.io/picard), and indexed the reference using SAMtools (Li et al. 2009). We then aligned all our samples read data to the reference using BWA-MEM (Li 2013), and the output SAM files were converted into BAM format using SAMtools. We marked duplicate reads to ensure that all our fragments had been independently targeted and merged all the individual BAM files into a single file using Picard. We used GATK 4.3.0.0 (McKenna et al. 2010) to call variants using HaplotypeCaller, extract SNPs and indels with SelectVariants, and filtered and extracted the passing variants (QUAL < 30.0, QD < 5.0) with VariantFiltration and SelectVariants. We outputted the passing SNPs into a VCF file and removed individuals that were missing more than 75% of the SNPs and all the loci that were missing in more than 50% of the individuals, resulting in 11,189 SNPs. We also created a second VCF file by selecting one random SNP per locus, resulting in 427 SNPs.

References

Blumenstiel B., Cibulskis K., Fisher S., DeFelice M., Barry A., Fennell T., Abreu J., Mine B., Costello M., Young G., Maguire J., Kernytsky A., Melnikov A., Rogov P., Gnirke A., Gabriel S. 2010. Targeted exon sequencing by in-solution hybrid selection. Curr. Protoc. Hum. Genet. 66:18.4.1-18.4.24.

Bolger A.M., Lohse M., Usadel B. 2014. Trimmomatic: a flexible trimmer for Illumina sequence data. Bioinformatics. 30:2114–20.

Brawand D., Wagner C.E., Li Y.I., Malinsky M., Keller I., Fan S., Simakov O., Ng A.Y., Lim Z.W., Bezault E., Turner-Maier J., Johnson J., Alcazar R., Noh H.J., Russell P., Aken B., Alföldi J., Amemiya C., Azzouzi N., Baroiller J.-F., Barloy-Hubler F., Berlin A., Bloomquist R., Carleton K.L., Conte M.A., D’Cotta H., Eshel O., Gaffney L., Galibert F., Gante H.F., Gnerre S., Greuter L., Guyon R., Haddad N.S., Haerty W., Harris R.M., Hofmann H.A., Hourlier T., Hulata G., Jaffe D.B., Lara M., Lee A.P., MacCallum I., Mwaiko S., Nikaido M., Nishihara H., Ozouf-Costaz C., Penman D.J., Przybylski D., Rakotomanga M., Renn S.C.P., Ribeiro F.J., Ron M., Salzburger W., Sanchez-Pulido L., Santos M.E., Searle S., Sharpe T., Swofford R., Tan F.J., Williams L., Young S., Yin S., Okada N., Kocher T.D., Miska E.A., Lander E.S., Venkatesh B., Fernald R.D., Meyer A., Ponting C.P., Streelman J.T., Lindblad-Toh K., Seehausen O., Di Palma F. 2014. The genomic substrate for adaptive radiation in African cichlid fish. Nature. 513:375–381.

Castresana J. 2000. Selection of conserved blocks from multiple alignments for their use in phylogenetic analysis. Mol. Biol. Evol. 17:540–552.

Faircloth B.C. 2013. illumiprocessor: a trimmomatic wrapper for parallel adapter and quality trimming. Available from http://dx.doi.org/10.6079/J9ILL.

Faircloth B.C. 2016. PHYLUCE is a software package for the analysis of conserved genomic loci. Bioinformatics. 32:786–788.

Faircloth B.C., Glenn. 2012. Not all sequence tags are created equal: Designing and validating sequence identification tags robust to indels. PLoS One. 7:e42543.

Faircloth B.C., Sorenson L., Santini F., Alfaro M.E. 2013. A phylogenomic perspective on the radiation of ray-finned fishes based upon targeted sequencing of ultraconserved elements (UCEs). PLoS One. 8:e65923.

Grabherr M.G., Haas B.J., Yassour M., Levin J.Z., Thompson D.A., Amit I., Adiconis X., Fan L., Raychowdhury R., Zeng Q., Chen Z., Mauceli E., Hacohen N., Gnirke A., Rhind N., di Palma F., Birren B.W., Nusbaum C., Lindblad-Toh K., Friedman N., Regev A. 2011. Full-length transcriptome assembly from RNA-Seq data without a reference genome. Nat. Biotechnol. 29:644–52.

Katoh K., Standley D.M. 2013. MAFFT multiple sequence alignment software version 7: improvements in performance and usability. Mol. Biol. Evol. 30:772–780.

Li H. 2013. Aligning sequence reads, clone sequences and assembly contigs with BWA-MEM. arXiv. arXiv:1303.3997.

Li H., Handsaker B., Wysoker A., Fennell T., Ruan J., Homer N., Marth G., Abecasis G., Durbin R., Subgroup. 1000 Genome Project Data Processing. 2009. The sequence alignment/map format and SAMtools. Bioinformatics. 25:2078–2079.

Marçais G., Kingsford C. 2011. A fast, lock-free approach for efficient parallel counting of occurrences of k-mers. Bioinformatics. 27:764–770.

McKenna A., Hanna M., Banks E., Sivachenko A., Cibulskis K., Kernytsky A., Garimella K., Altshuler D., Gabriel S., Daly M., DePristo M. 2010. The genome analysis toolkit: a MapReduce framework for analyzing next-generation DNA sequencing data. Genome Res. 20:1297–1303.

Rohland N., Reich R. 2012. Cost-effective, high-throughput DNA sequencing libraries for multiplexed target capture. Genome Res. 22:939–946.

**Figure S1.** Maximum Likelihood phylogenetic hypotheses of Lamprologini based on mitochondrial gene ND2 sequences using IQ-TREE2.

**Figure S2.** Bayesian Inference phylogenetic hypotheses of Lamprologini based on mitochondrial gene ND2 sequences using MrBayes.

**Figure S3.** Local posterior probability, normalized quartet score, and number of informative gene trees for the main and alternative quartet topologies for all the nodes in the Congo River *Lamprologus* clade as recovered in ASTRAL-III. N1-N10 correspond to the nodes in the small inset tree at the top.

**Figure S4.** Average Delta scores ± SD for all the species of riverine *Lamprologus* included in the SplitsTree analysis.

**Figure S5.** Haplotype network of the “mainly LCR” clade samples of riverine *Lamprologus* based on mitochondrial gene ND2 sequences using PopArt.

 **Figure S6.** Haplotype network of the “mainly CUCR” clade samples of riverine *Lamprologus* based on mitochondrial gene ND2 sequences using PopArt.

**Figure S7.** Calibrated timetree of Lamprologini based on the fixed ML topology using BEAST2. Values indicate mean age estimates and bars represent 95% HPD intervals for each node. The estimates are the combined result of four independent analyses of ~100 UCE loci ran by duplicate.

**Figure S8. Cross-entropy values** as inferred from sNMF to choose the best-fitting (lowest) *K* value for our data values of *K*.

**Figure S9.** Ancestry coefficients of riverine *Lamprologus* species as inferred from sNMF for the most frequent structure arrangements recovered from *K* = 2 through K = 10. Each bar represents an individual sample.

**Figure S10.** Polytomy test results for three datasets using all available gene trees with nodes collapsed at increasing thresholds of bootstrap support (BS) a) all nodes included, b) nodes with BS>1 included, c) nodes with BS>10 included. In the x-axis we show, for each internal branch in the ASTRAL species tree, its estimated length in coalescent units (CU) in log scale, and in the y-axis the polytomy test 1-*p*-value and the local posterior probability (LPP) estimated in the species tree. The dashed line indicates the significance level (1-*p*-value = 0.95) of the polytomy test.


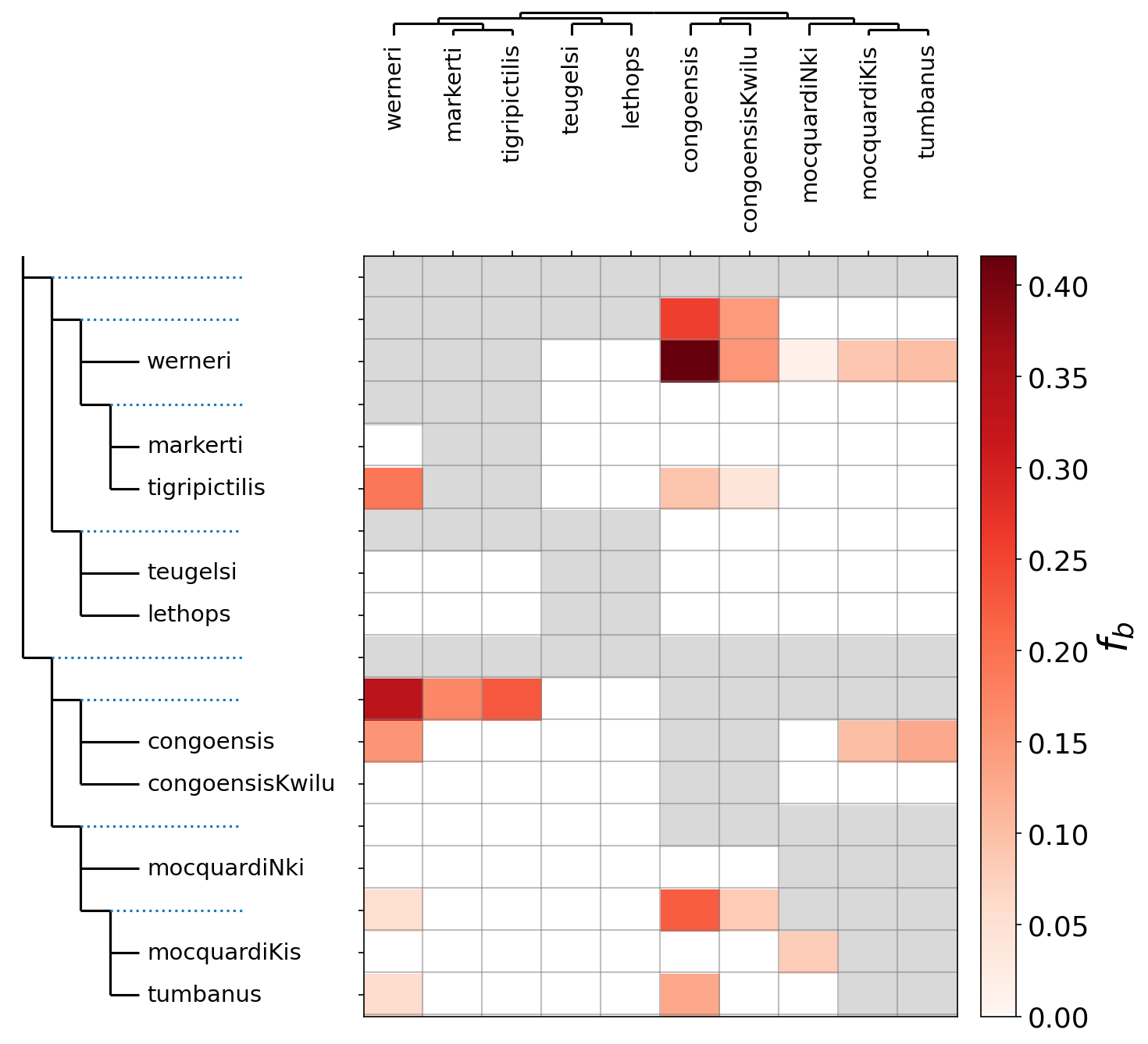


**Figure S11.** *f*-branch test results for the ASTRAL tree topology.


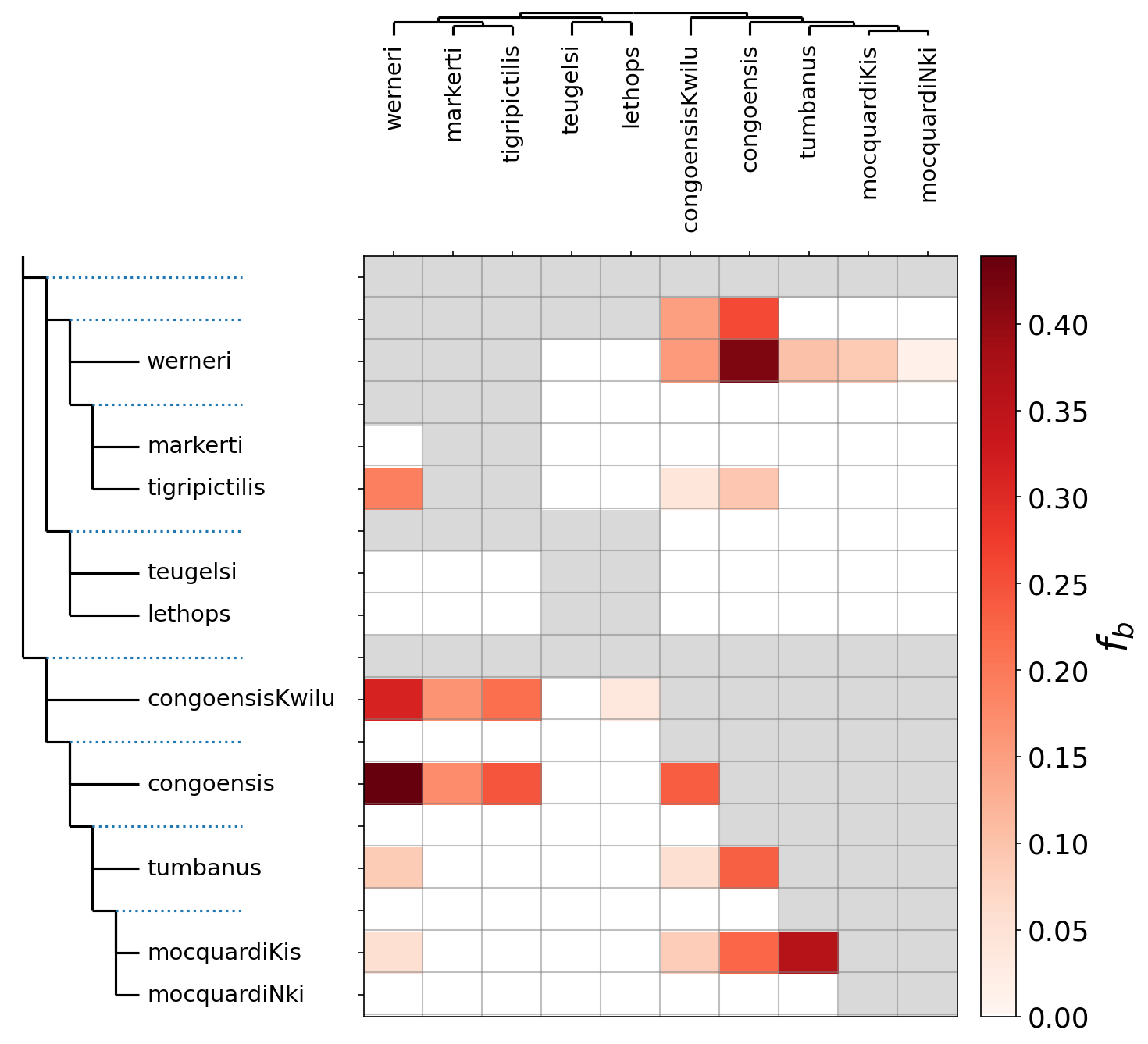


**Figure S12.** *f*-branch test results for the ML tree topology.

**Figure S13.** Calibrated timetree of mitochondrial ND2 gene sequences of Lamprologini using BEAST2. Values indicate mean age estimates and bars represent 95% HPD intervals for each node. Samples in red represent the “mainly LCR” clade and samples in blue represent the “mainly CUCR” clade.
